# Modelling the Impact of NETosis During the Initial Stage of Systemic Lupus Erythematosus

**DOI:** 10.1007/s11538-024-01291-3

**Published:** 2024-04-28

**Authors:** Vladimira Suvandjieva, Ivanka Tsacheva, Marlene Santos, Georgios Kararigas, Peter Rashkov

**Affiliations:** 1 Institute of Mathematics and Informatics, Bulgarian Academy of Sciences, ul. Akad. Georgi Bonchev, blok 8, 1113 Sofia, Bulgaria; 2 Faculty of Biology, Sofia University “Sveti Kliment Ohridski”, bul. Dragan Tsankov 8, 1164 Sofia, Bulgaria; 3 LAQV/REQUIMTE, Escola Superior de Saúde, Instituto Politécnico do Porto, Rua Dr. António Bernardino de Almeida, 400, 4200-072 Porto, Portugal; 4 Department of Physiology, Faculty of Medicine, University of Iceland, Vatnsmyrarvegur 16, 101 Reykjavik, Iceland

**Keywords:** Lupus, Immune response, Bifurcation analysis, Antigen presentation, 92C30, 34A34, 34C23

## Abstract

The development of autoimmune diseases often takes years before clinical symptoms become detectable. We propose a mathematical model for the immune response during the initial stage of Systemic Lupus Erythematosus which models the process of aberrant apoptosis and activation of macrophages and neutrophils. NETosis is a type of cell death characterised by the release of neutrophil extracellular traps, or NETs, containing material from the neutrophil’s nucleus, in response to a pathogenic stimulus. This process is hypothesised to contribute to the development of autoimmunogenicity in SLE. The aim of this work is to study how NETosis contributes to the establishment of persistent autoantigen production by analysing the steady states and the asymptotic dynamics of the model by numerical experiment.

**Supplementary Information:**

The online version contains supplementary material available at 10.1007/s11538-024-01291-3.

## Introduction

A pathogenic adaptive immune response manifests itself in terms of autoimmunity or chronic inflammation, but just like the normal immune response, it relies on prior activation of the innate immunity. If the innate response is excessive or protracted in time, it may trigger a pathogenic adaptive response, especially in genetically predisposed patients (Theofilopoulos et al. 2011; Tsokos et al. 2016).

Aberrant apoptosis and dysfunctional clearance of biological waste are associated with the emergence of autoimmune response in systemic lupus erythematosus (SLE). In the process of programmed cell death, chromatin from the nucleus is translocated to the cell surface in microvescicles or blebs. Under normal circumstances, early apoptotic cells are cleared from the tissue by macrophages and dendritic cells, without causing inflammation.

When this process is disrupted, apoptotic blebs at the surface of a dying cell may start to break and nuclear material which has accumulated inside them could be spontaneously released in the tissue (Casciola-Rosen et al. 1994) and exposed to the immune system. Since cellular content has been partially modified during the apoptotic process (resulting, for instance, in modified histones, chromatin), it may escape the normal tolerogenic mechanism, become immunogenic and initiate an aberrant immune response, involving abnormal T and B lymphocyte activation, pro-inflammatory signalling, and production of broad spectrum of autoantibodies (Dieker et al. 2007; Fransen et al. 2010; Tsokos et al. 2016; Yaniv et al. 2015). A hallmark of SLE is the production of autoantibodies against components of nuclear origin, including chromatin, and the deposition of chromatin-antibody complexes in tissue exacerbates local inflammation and organ damage (Dieker et al. 2015). The process of the spread of autoimmunity may last for years before the onset of clinical symptoms, and the mechanisms behind the build-up and estabishment of persistent autoantigen production during the initiation stage of SLE are not entirely clear (Tsokos et al. 2016; Tsokos 2020).

Neutrophils are white blood cells (leukocytes) which make up to $70\%$ of the white blood cells in the human body (Okada et al. 2010). They play a critical role in the innate immune response by fighting pathogens. Along with phagocytosis and degranulation, another weapon in the neutrophil arsenal for fighting pathogens is NETosis, a process based on expelling chromatin, nuclear, cytoplasmic and granular material, proinflammatory cytokines, and antimicrobial peptides from the neutrophil cell, resulting in its death and the formation of a neutrophil extracellular trap (NET) (Smith and Kaplan 2015). The NET is made of decondensed chromatin, forming web-like DNA structures whose role is to trap pathogens and to prevent their further spread in the organism (Gillot et al. 2021).

With increased understanding of the mechanism of NETosis, it has been nicknamed a “double-edged” sword (Thiam et al. 2020), since the presence of NETs may also be associated with an inadequate immune response (Gillot et al. 2021). The reason is that exposed, extracellular chromatin could be recognised as an antigen, triggering an immunogenic response against the host organism itself. NETosis is suspected to be a factor for the development of autoimmune diseases, such as SLE (de Bont et al. 2019; Thiam et al. 2020), and for complications in infectious diseases including blood clots in severe forms of COVID-19 (Gillot et al. 2021), which may also occur in a sex-biased manner (Ritter and Kararigas 2020; Kararigas 2022).

Defective clearance of biological waste (apoptotic cells, nuclear debris, immune complexes, NETs) following an environmental trigger, an infection, injury, stress or trauma is an important factor for the emerging loss of tolerance, initiation of an autoimmune response and tissue damage in SLE (Gaipl et al. 2005; Tsokos et al. 2016). Circulating chromatin with apoptotic origin in serum is associated with SLE and is absent from serum of patients with rheumatoid arthritis and systemic sclerosis (Dieker et al. 2016). On one hand, microparticles derived from apoptotic cells in the case of SLE have been found to enhance the formation of NETs, leading to a feed-forward effect on the autoimmune response (Dieker et al. 2016; Villanueva et al. 2011). On the other hand, neutrophils in SLE patients’ serum are more prone to NET formation, serving as a source of autoantigen themselves (Bouts et al. 2012).

In this study, we present a novel mathematical model of the basic interactions between the major players in NETosis: neutrophils and macrophages (M$\Phi$), which are antigen-presenting cells (APCs). We consider two types of antigen: material originating from apoptosis which does not elicit inflammation, and autoantigen with diverse origins such as content of apoptotic blebs that have ruptured and expose modified nuclear material, such as chromatin, as well as nuclear content expelled from neutrophils during NETosis.

We use this mathematical model to study the contribution of one innate immune mechanism, NETosis, to the complex process in the SLE initiation stage. We perform theoretical analysis and conduct numerical experiments to identify conditions that lead to persistence of autoantigen in the organism. This event is important for SLE pathophysiology whereby it could cause inflammation and, over time, initiate an adaptive autoimmune response process, for instance, after autoantigen delivery to the lymph node. This distinguishes our work from models in the literature which focus on the chronic stage of SLE and assume an established autoimmune response (Budu-Grajdeanu et al. 2010; Gao et al. 2022), study organ damage in the case of Lupus nephritis (Hao et al. 2014), or work with aggregate features of the disease without elucidating the mechanisms behind its pathophysiology (Yazdani et al. 2023).

In Sect. 2, we present the model described as a system of ordinary differential equations. In Sect. 3, we analyse the steady states branches of the model which have biological relevance, and the type of bifurcations that connect them. Section 4 summarises the numerical experiments conducted to analyse the bifurcation structure where it is not possible to derive analytical results, and some examples of the temporal dynamics. We conclude in Sect. 5 with a discussion of the model’s properties and their biological interpretation. The model reveals that several types of equilibria are possible, which correspond to a normal and pathological states. The dynamics can exhibit bistability as well as oscillatory regime for various parameter ranges. These features support the important, but not exclusive role of NETosis in the pathogenesis of SLE, but also show that macrophage activity is important in the accumulation of apoptotic waste and autoantigen.

## Mathematical Model

We consider a simple scheme of interactions reflecting the production of apoptotic material $x_1$, autoantigen (modified and exposed nuclear and cytoplasmic material, also known as exposed hidden-self in the literature) $x_2$, and two types of cells: neutrophils *z*(*t*) and antigen-presenting cells *y*(*t*) (macrophages, M$\Phi$) which are activated and recruited by the presence of either types of antigen. The APCs produce proinflammatory cytokines, which in turn activate neutrophils. We study how the interactions between them could lead to a sustained production of autoantigen that is a hallmark of the initial stages of Lupus, whereby a dysfunctional immune response could arise as a result of an environmental trigger, a pathogen infection or tissue damage [e.g. due to UV irradiation or exposure to toxins (Tsokos et al. 2016)].

**Table 1:** Phase variables of the model with their units

Variable	Definition	Unit
$x_1(t)$	Apoptotic material	$\upmu$g/ml
$x_2(t)$	Autoantigen	$\upmu$g/ml
*y*(*t*)	Macrophages	cells/ml
*z*(*t*)	Neutrophils	cells/ml

**Fig. 1:**
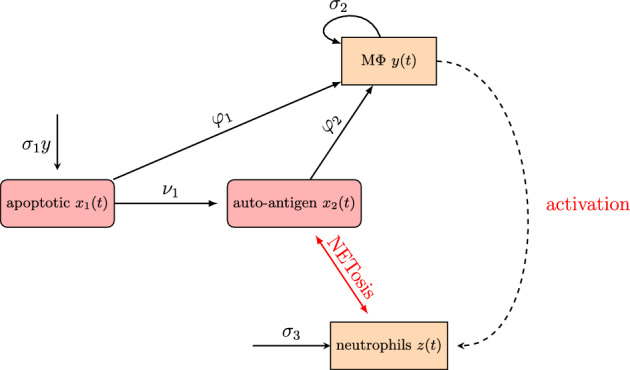
Scheme of the model

The variables with their units are listed in Table 1, a scheme of the model is given in Fig. 1, and the system of ordinary differential equations describing the dynamics is in (1):

1a $$\begin{aligned} x_1'&=\sigma _1 y-\frac{\beta _1 y x_1}{\kappa _y+x_1+x_2}-\nu _1 x_1-\mu _1 x_1 \end{aligned}$$

1b $$\begin{aligned} x_2'&= \nu _1 x_1-\frac{\beta _2 y x_2}{\kappa _y+x_1+x_2}+\alpha \nu _2 z x_2-\mu _2 x_2 \end{aligned}$$

1c $$\begin{aligned} y'&=\biggl (\frac{\beta _1 y x_1}{\kappa _y+x_1+x_2}+\frac{\beta _2 y x_2}{\kappa _y+x_1+x_2} \biggl ) \sigma _2-\mu _4 y-\mu _5 y^2 \end{aligned}$$

1d $$\begin{aligned} z'&=\sigma _3+\frac{\beta _3 y}{\kappa _z+y}-\mu _3 z-\nu _2 z x_2 \end{aligned}$$

The equation for apoptotic material (1a) contains a term for its production which depends on the macrophages $\sigma _1y$. This is a simple way to model the very complex process of apoptosis, which involves an intricate network of chemokines, cytokines and immune cells. While macrophages induce apoptosis in normal cells in vivo (Diez-Roux and Lang 1997; Lang and Bishop 1993), they also trigger production of pro-inflammatory cytokines and chemokines (such as IL-6, IL-12, IL-18, TNF$\alpha$). These activate dendritic cells and killer T-cells, whose cytotoxic action causes apoptosis in situ (Vermare et al. 2022).

The remaining terms in (1a) represent the removal of apoptotic material by macrophages at rate $\varphi _1y$, and due to other factors at rate $\mu _1x_1$ (such as action of the complement system) that we do not model explicitly. Apoptotic material $x_1$ that is not picked up and cleared by macrophages action eventually converts into late apoptotic material in blebs at rate $\nu _1x_1$. The functional form of this conversion process is well-known from ecological models of predation with resource conversion (Focardi et al. 2017; Jansen and Van Gorder 2018; Nevai and Van Gorder 2012), and has been used as well as in a model for type I diabetes (Marée et al. 2006).

The terms for autoantigen production in (1b) are two: one represents the quantity originating from material in blebs $\nu _1x_1$, and another for the amount of nuclear and cytoplasmic material released as a result of NET formation. The term for the NET formation in (1b) follows the law of mass action, and is proportional to the quantities of neutrophils and autoantigen—it occurs at a rate $\alpha \nu _2x_2z$. Here, the parameter $\alpha$ represents the yield of autoantigen during NETosis, an important source of the nuclear antigens that cause auto-antibody production in SLE patients (Lande et al. 2011; Tsokos et al. 2016). autoantigen is picked up by macrophages at rate $\varphi _2 y$ and removed due to other factors such as complement at rate $\mu _2x_2$.

The picking and removal of antigen by macrophages is modelled with a motivation in ecological models of consumers with multiple resources (Abrams 1987; Marten 1973). We use a Holling type-II functional response with competition:

$$\begin{aligned} \varphi _1(x_1,x_2)=\frac{\beta _1 x_1}{\kappa _y+x_1+x_2},\quad \varphi _2(x_1,x_2)=\frac{\beta _2 x_2}{\kappa _y+x_1+x_2}. \end{aligned}$$

This particular functional response represents the assumption that an individual antigen-presenting cell is constrained in its capacity to pick up circulating antigen, and to internalise it, processing it into the peptide fragments which are displayed on its membrane. Hence, $\varphi _i(x_1,x_2)$ is a increasing but saturating function in $x_i,i=1,2$, and reflects the constraint of pick-up and internalisation as in Holling’s original model from ecology (Holling 1959). However, handling the other circulating antigen $x_{3-i}$ reduces the APC capacity to pick up and process $x_i$. Due to the competition between the two types of circulating antigen in the model, the functional response $\varphi _i$ is a decreasing function of $x_{3-i}$. In vitro observations of digestion of apoptotic cells by macrophages in animals with autoimmune diabetes (Marée et al. 2005) also supports the use of such functional response in the model.

The equation for activated macrophages (1c) contains a term for their activation and recruitment after uptake of the two types of material: $x_1$ being apoptotic in origin, but not inflammatory, and $x_2$ being autoimmunogenic stemming from nuclear material in late apoptotic blebs or resulting from NETosis. This is represented by a bulk rate $\sigma _2$ to keep the model structure simple enough. The quadratic term $-\mu _4 y-\mu _5 y^{2}$ representing the macrophages’ growth towards a carrying capacity $\frac{(\beta _1+\beta _2)\sigma _2-\mu _4}{\mu _5}$ in the presence of antigen. In this manner the model takes into account crowding effects that reduce the growth of the activated macrophages population as done in a model for type-1 diabetes (Marée et al. 2006).

The equation for neutrophils (1d) contains constant production term $\sigma _3$ and degradation with rate $\mu _3$ term. The last term stands for NET formation, as already mentioned. The term $\frac{\beta _3 y}{\kappa _z+y}$ accounts for the stimulatory action of proinflammatory cytokines resulting from the action of macrophages. In fact, type I interferon primes neutrophils for NET release in patients with SLE, suggesting a possible positive feedback loop (Lande et al. 2011; Tsokos et al. 2016).

Note that the model (1) does not distinguish between macrophages which belong to type M1 (classically activated) or M2 (alternatively activated). The reason for this is to maintain the structure simple enough, especially because we do not explicitly model any pro-inflammatory cytokines, nor presentation of antigens to B cells which leads to production of autoantibodies in the longer run. We stress that strict positivity of $A=(\beta _1+\beta _2)\sigma _2-\mu _4$ is required in (1c) because otherwise $y'(t)$ will always be negative.

Parameter description and value ranges used in the model and in the numerical simulations are listed in Table 2 and under the respective figures illustrating the findings. The parameter range estimates are provided in the Supplementary Material.

In Sect. 3, we perform analysis of the equilibria of (1). We show that this model has stable equilibria with strictly positive amounts of autoantigen $x_2$, as well as stable limit cycles where the two types of antigen coexist in time. In addition, the model also demonstrates multistationarity and bistability, as illustrated by numerical bifurcation analysis in Sect. 4.

Estimates for the analytical solution of (1) (non-negativity and uniform boundedness in time) are made for $t>0$. Their proofs are included as Propositions 1 and 2 in the Supplementary Material. These statements together imply that the solution to (1) exists for all $t>0$ for non-negative initial conditions.

**Table 2:** Parameter definitions and values used in the numerical experiments

Parameter	Definition	Value/Range	Unit	Reference
$\alpha$	Yield of autoantigen from NETosis	$[0,2.5{\times }10^{-4}]$	$\upmu$g/cell	Estimated
$\beta _1$	Maximum pick-up rate of apoptotic material by macrophages	$0.002~[0.5{\times }10^{-4},4.05{\times }10^{-3}]$	$\upmu$g/(cell day)	Estimated
$\beta _2$	Maximum pick-up rate of autoantigen by macrophages	$0.002~[0.5{\times }10^{-4},4.05{\times }10^{-3}]$	$\upmu$g/(cell day)	Estimated
$\beta _3$	Maximum activation rate of neutrophils by macrophages	As indicated	cells/(ml day)	Estimated
$\kappa _y$	Michaelis constant, pick-up rate of antigen by macrophages	1	$\upmu$g/ml	Guess
$\kappa _z$	Michaelis constant, activation rate of neutrophils by macrophages	$10^4$	cells/ml	Guess
$\mu _1$	Removal rate of apoptotic material	[10, 12]	day^-1^	Estimated
$\mu _2$	Removal rate of autoantigen	[11, 12]	day^-1^	Estimated
$\mu _3$	Removal rate of neutrophils	[0.86, 1.28]	day^-1^	Patel et al. (2021)
$\mu _4$	Removal rate of macrophages	0.2	day^-1^	Marée et al. (2006), Patel et al. (2021)
$\mu _5$	Crowding coefficient, macrophages	As indicated	(cells day)^-1^	Estimated
$\nu _1$	Production rate of late apoptotic material (blebs)	0.05, 0.5	day^-1^	van Nieuwenhuijze et al. (2003)
$\nu _2$	NETosis rate	As indicated	($\upmu$g/ml day)^-1^	Guess
$\sigma _1$	Production rate of apoptotic material	$10^{-5}$	$\upmu$g/(cell day)	Guess
$\sigma _2$	Activation/recruitment rate of macrophages	As indicated	cells/$\upmu$g	Estimated
$\sigma _3$	Production of neutrophils	$[3.7,5]{\times }10^6$	cells/(ml day)	Patel et al. (2021), Tatsukawa et al. (2008)

## Analysis of Equilibria

We study the equilibria (steady states) of the model by setting the right-hand side of (1) to zero and solving the corresponding algebraic system. For every equilibrium, we analyse the local asymptotic stability using the Jacobi matrix of the right-hand side of system (1) We distinguish between three types of equilibrium points which have biological relevance:

1. *Normal state* $E_0$ with $x_1=x_2=y=0, z=\frac{\sigma _3}{\mu _3}$. In this equilibrium, there is no apoptosis, no antigen and no activated macrophages, and neutrophils are at equilibrium. The Jacobi matrix is
  2 $$\begin{aligned} \begin{aligned} \textbf{J}(E_0)= \begin{pmatrix} -\nu _1-\mu _1 &{} \quad 0 &{} \quad \sigma _1 &{} \quad 0 \\ \nu _1 &{} \quad \alpha \nu _2 \frac{\sigma _3}{\mu _3}-\mu _2 &{} \quad 0 &{} \quad 0\\ 0 &{} \quad 0 &{} \quad -\mu _4 &{} \quad 0\\ 0 &{} \quad -\nu _2 \frac{\sigma _3}{\mu _3} &{} \quad \frac{ \beta _3}{\kappa _z} &{} \quad 0 \end{pmatrix} \end{aligned} \end{aligned}$$
  The eigenvalues of the Jacobi matrix in this case are
  $$\begin{aligned} \lambda _1=-\mu _3, \lambda _2=-\mu _4, \lambda _3=-\nu _1-\mu _1, \lambda _4=\frac{\alpha \sigma _3 \nu _2-\mu _2 \mu _3}{\mu _3}, \end{aligned}$$
  and the equilibrium $E_0$ is locally asymptotically stable when the parameters of the model satisfy the inequality $\alpha \sigma _3 \nu _2-\mu _2 \mu _3<0$, i.e.
  3 $$\begin{aligned} \alpha <\alpha _0=\frac{\mu _2 \mu _3}{\sigma _3 \nu _2}. \end{aligned}$$
2. *Absence of apoptosis state* $E_1$, with equilibrium components
  $$\begin{aligned} x_1=y=0, x_2=\frac{\alpha \sigma _3 \nu _2-\mu _3 \mu _2}{\mu _2 \nu _2}, z=\frac{\mu _2}{\alpha \nu _2}. \end{aligned}$$
  In $E_1$ there are no activated macrophages, and no material with apoptotic origin, while the only positive components are the neutrophils and the autoantigen resulting from NETosis only. This state would represent a pathological state of the immune system. $E_1$ is feasible so long as $\alpha \sigma _3 \nu _2-\mu _3 \mu _2>0$, i.e.
  4 $$\begin{aligned} \alpha >\alpha _0=\frac{\mu _2 \mu _3}{\sigma _3 \nu _2}. \end{aligned}$$
  The Jacobi matrix is
  5 $$\begin{aligned} \textbf{J}(E_1)= \begin{pmatrix} -\nu _1-\mu _1 &{} \quad 0 &{} \quad \sigma _1 &{} \quad 0 \\ \nu _1 &{} \quad 0 &{} \quad \beta _2 \biggl (\frac{\kappa _y \mu _2 \nu _2}{-\mu _2 \mu _3+\kappa _y \mu _2 \nu _2+\alpha \nu _2 \sigma _3}-1\biggl ) &{} \quad \alpha \biggl (\frac{\alpha \nu _2 \sigma _3}{\mu _2}-\mu _3\biggl )\\ 0 &{} \quad 0 &{} \quad \beta _2 \sigma _2 \biggl (1-\frac{\kappa _y \mu _2 \nu _2}{-\mu _2 \mu _3+\kappa _y \mu _2 \nu _2+\alpha \nu _2 \sigma _3}\biggl )-\mu _4 &{} \quad 0\\ 0 &{} \quad -\frac{\mu _2}{\alpha } &{} \quad \frac{ \beta _3}{\kappa _z} &{} \quad -\frac{\alpha \nu _2 \sigma _3}{\mu _2} \end{pmatrix}.\nonumber \\ \end{aligned}$$
  The eigenvalues of the Jacobi matrix $\textbf{J}(E_1)$ are
  $$\begin{aligned} \lambda _1&=-\mu _1-\nu _1,\\ \lambda _2&=\beta _2 \sigma _2 \biggl (1-\frac{k_2 \mu _2 \nu _2}{k_2 \mu _2 \nu _2+\alpha \nu _2 \sigma _3-\mu _2 \mu _3} \biggl )-\mu _4, \\ \lambda _3&= p+q, \lambda _4=p-q \end{aligned}$$
  where *p* and *q* depend on the parameters of the model. Moreover, $\lambda _3+\lambda _4=-\frac{\alpha \nu _2 \sigma _3}{\mu _2}<0$ and $\lambda _3\lambda _4=\alpha \sigma _3 \nu _2-\mu _2 \mu _3>0$ because otherwise the $x_2$ component of the equilibrium point $E_1$ would be negative. The last two estimates together mean that $\lambda _3$ and $\lambda _4$ are either both negative real or complex conjugates with negative real parts. Therefore, the only condition for the (local) stability of $E_1$ is $\lambda _2<0$ which depends on the choice of the parameters and requires
  6 $$\begin{aligned} \alpha <\alpha _1=\frac{\mu _2 \mu _3 \mu _4-\kappa _y \mu _2 \mu _4 \nu _2-\beta _2 \mu _2 \mu _3 \sigma _2}{\mu _4 \nu _2 \sigma _3-\beta _2 \nu _2 \sigma _2 \sigma _3}. \end{aligned}$$
  This condition is compatible with the feasibility condition (4) stated above because $\alpha _0<\alpha _1$ for every choice of parameters. $E_1$ may actually be never observed in vivo because, in practice, macrophages would be always recruited to a site of inflammation, and some apoptosis would occur there. It may so happen that in our simplified model, the system may converge asymptotically to $E_1$, a state where autoantigen production resulting from NETosis would persist after clearance of apoptotic material. For the parameter values we choose for the bifurcation analysis, the range of $\alpha$ where $E_1$ is locally asymptotically stable is very narrow.
3. *Coexistence of antigen state* $E_*$ (strictly positive quantities of all phase variables, $x_1^*,x_2^*,y^*,z^*>0$). This equilibrium represents the onset of pathology where autoantigen persists as a result of insufficient clearance of apoptotic material becoming exposed to the immune system in the form of ruptured apoptotic blebs or from nuclear or cytoplasmic content expelled from neutrophils during NETosis. Computation of the exact values involves solutions of high-degree polynomial whose explicit solution is not feasible.

### Bifurcations at the Threshold Values

Bifurcation theory gives dependence of qualitative model outputs upon variation of some or more parameter values. To examine the type of bifurcations at the threshold values of $\alpha$ between $E_0,E_1$ and $E_1,E_*$ we use Sotomayor’s theorem (Perko 2001). Let us denote the right hand-side of (1) by

$$\begin{aligned} \textbf{f}(x_1, x_2, y, z, \alpha )= \begin{pmatrix} \sigma _1 y-\frac{\beta _1 y x_1}{\kappa _y+x_1+x_2}-\nu _1 x_1-\mu _1 x_1 \\ \nu _1 x_1-\frac{\beta _2 y x_2}{\kappa _y+x_1+x_2}+\alpha \nu _2 z x_2-\mu _2 x_2 \\ \biggl (\frac{\beta _1 y x_1}{\kappa _y+x_1+x_2}+\frac{\beta _2 y x_2}{\kappa _y+x_1+x_2} \biggl ) \sigma _2-\mu _4 y-\mu _5 y^2 \\ \sigma _3+\frac{\beta _3 y}{\kappa _z+y}-\mu _3 z-\nu _2 z x_2 \end{pmatrix}. \end{aligned}$$

Using the estimates from the previous section we set as bifurcation parameter $\alpha$ and threshold values

$$\begin{aligned} \alpha _0&=\frac{\mu _2 \mu _3}{\sigma _3 \nu _2},\\ \alpha _1&=\frac{\mu _2 \mu _3 \mu _4-\kappa _y \mu _2 \mu _4 \nu _2-\beta _2 \mu _2 \mu _3 \sigma _2}{\mu _4 \nu _2 \sigma _3-\beta _2 \nu _2 \sigma _2 \sigma _3}. \end{aligned}$$

The partial derivative of $\textbf{f}$ with respect to $\alpha$ is

$$\begin{aligned} \textbf{f}_{\alpha }(x_1, x_2, y, z, \alpha )=\frac{\partial \textbf{f}(x_1, x_2, y, z, \alpha )}{\partial \alpha }=(0,x_2 z \nu _2,0,0)^{T}. \end{aligned}$$

In the following we shall present some analysis of bifurcations of the model as we vary the parameter $\alpha$.

#### Transcritical Bifurcation Between $E_0$ and $E_1$

It is already shown in the previous section that $\textbf{J}(E_0)$ has three strictly negative eigenvalues. The only eigenvalue of $\textbf{J}(E_0)$ that can become zero is $\lambda _4=\frac{\alpha \sigma _3 \nu _2-\mu _2 \mu _3}{\mu _3}$ exactly when $\alpha =\alpha _0$. Let $\textbf{v}_0$ be the right eigenvector corresponding to the zero eigenvalue $\lambda _4=0$ of $\textbf{J}(E_0)$ in this case,

$$\begin{aligned} \textbf{v}_0=\left( 0,-\frac{\mu _3^{2}}{\nu _2 \sigma _3},0,1\right) ^T. \end{aligned}$$

Let $\textbf{w}_0$ be the right eigenvector corresponding to $\lambda _4=0$ of $\textbf{J}^\textrm{T}(E_0)$,

$$\begin{aligned} \textbf{w}_0^T=\left( \frac{\mu _4}{\sigma _1},\frac{\mu _4(\mu _1+\nu _1)}{\nu _1 \sigma _1},1,0\right) . \end{aligned}$$

Then

$$\begin{aligned}&\textbf{w}_0^{T} f_{\alpha } \left( 0,0,0,\frac{\sigma _3}{\mu _3},\alpha _0 \right) =\frac{x_2 z \mu _4(\mu _1+\nu _1) \nu _2}{\nu _1 \sigma _1}=0 ,\\&\textbf{w}_0^{T} \left[ \textbf{D} f_{\alpha } \left( 0,0,0,\frac{\sigma _3}{\mu _3},\alpha _0 \right) \textbf{v}_0 \right] =-\frac{\mu _3 \mu _4 (\mu _1+\nu _1)}{\nu _1 \sigma _1}<0,\\&\textbf{w}_0^{T} \left[ \textbf{D}^{2}f \left( 0,0,0,\frac{\sigma _3}{\mu _3},\alpha _0 \right) \left( \textbf{v}_0,\textbf{v}_0 \right) \right] =-\frac{2 \mu _2 \mu _3 \mu _4(\mu _1+\nu _1)}{\nu _1 \nu _2 \sigma _1 \sigma _3^{2}}<0. \end{aligned}$$

Then Sotomayor’s theorem implies that the system (1) experiences a transcritical bifurcation at $E_0$ as $\alpha$ varies through the threshold value $\alpha _0$.

#### Bifurcation Between $E_1$ and $E_*$

It is clear from the previous section that the only eigenvalue of $\textbf{J}(E_1)$ which can vanish is $\lambda _2$ and this happens exactly when $\alpha =\alpha _1$. Let $\textbf{v}_1$ be the right eigenvector corresponding to the zero eigenvalue $\lambda _2=0$ of $\textbf{J}(E_1)$,

$$\begin{aligned} \textbf{v}_1= \begin{pmatrix} \frac{k_y \mu _2 \mu _4 \sigma _1 \sigma _2 ( k_y \mu _4 \nu _2 + \beta _2 \mu _3 \sigma _2-\mu _3 \mu _4 )}{(\mu _4 - \beta _2 \sigma _2)^{2} (\mu _4 (\mu _1 + \nu _1) - \nu _1 \sigma _1 \sigma _2) \sigma _3}\\ \frac{( k_y \mu _4 \nu _2 + \beta _2 \mu _3 \sigma _2-\mu _3 \mu _4)^{2} (k_y \beta _3 \mu _2 \mu _4 (\mu _1 + \nu _1) \sigma _2 + \kappa _z (\mu _4 - \beta _2 \sigma _2) (\mu _4 (\mu _1 + \nu _1) - \nu _1 \sigma _1 \sigma _2) \sigma _3)}{\kappa _z \nu _2 ( \beta _2 \sigma _2-\mu _4)^{3} (\mu _4 (\mu _1 + \nu _1) - \nu _1 \sigma _1 \sigma _2) \sigma _3^{2}}\\ \frac{k_y \mu _2 \mu _4 (\mu _1 + \nu _1) \sigma _2 ( \kappa _y \mu _4 \nu _2 + \beta _2 \mu _3 \sigma _2-\mu _3 \mu _4)}{(\mu _4 - \beta _2 \sigma _2)^{2} (\mu _4 (\mu _1 + \nu _1) - \nu _1 \ \sigma _1 \sigma _2) \sigma _3} \\ 1 \end{pmatrix}. \end{aligned}$$

Let $\textbf{w}_1$ be the left eigenvector corresponding to the zero eigenvalue $\lambda _2=0$ of $\textbf{J}(E_1)$,

$$\begin{aligned} \textbf{w}_1^T=(0,0,1,0). \end{aligned}$$

Then

$$\begin{aligned}&\textbf{w}_1^{T}f_{\alpha } \left( 0, \frac{\alpha _1 \sigma _3 \nu _2 - \mu _3 \mu _2}{\mu _2 \nu _2}, 0,\frac{\mu _2}{\alpha _1 \nu _2}, \alpha _1 \right) =0,\\&\textbf{w}_1^{T} \left[ D f_{\alpha } \left( 0, \frac{\alpha _1 \sigma _3 \nu _2 - \mu _3 \mu _2}{\mu _2 \nu _2}, 0,\frac{\mu _2}{\alpha _1 \nu _2}, \alpha _1 \right) v_1 \right] =0, \end{aligned}$$

which means that the Sotomayor’s theorem is inconclusive in this case, yet the transcritical bifurcation can be established by numerical continuation for the sets of parameter values we employ.

The analytic expressions for the threshold values $\alpha _0,\alpha _1$ reveal an inverse correlation with the rate of NETosis $\nu _2$. Whenever $\nu _2$ decreases, both $\alpha _0,\alpha _1$ increase. If other parameters are kept constant, one would expect that with a sufficient decrease in $\nu _2$, the normal state $E_0$ could become locally and, potentially, globally asymptotically stable over the entire biologically relevant range of $\alpha$. However, in Sect. 3.2 we show that in general, stability of $E_0$ could be at most local for $\alpha \approx 0$.

### Multistationarity for $\alpha \approx 0$

We show that the model (1) can exhibit the property of multistationarity for small values of $\alpha$. In other words we show that at least two equilibria of coexistence type $E^1_*\ne E^2_*$ may exist in parallel for $\alpha \approx 0$ depending on the choice of parameters. This is important because a dynamical system with multiple steady states may exhibit bistability. In other words, for a given set of parameter values the temporal evolution can have different asymptotic behaviour depending on the initial condition. In fact, we have already established that for sufficiently small $\alpha$, the normal state $E_0$ is locally asymptotically stable. If one of the coexistence equilibria for this range is also locally asymptotically stable, then the system (1) has the bistability property.

Let $\alpha =0$, and set the right-hand side of (1) to 0. Then the algebraic equation for $z'=0$ is uncoupled from the other three and we transform the equations for $x_1',x_2',y'=0$ algebraically to solve for the equilibrium values.

We assume $y\ne 0$ in order to divide $y'=0$ by *y*. Multiplying the resulting equation from (1c) by $\sigma _2$ and adding (1a) and (1b) to it yields

7 $$\begin{aligned} \mu _1 x_1+\mu _2 x_2=\sigma _1 y - \frac{\mu _4y}{\sigma _2} -\frac{\mu _5y^2}{\sigma _2}. \end{aligned}$$

On the other hand, rearranging (1c) gives

8 $$\begin{aligned} (\sigma _2\beta _1 -(\mu _4+\mu _5y))x_1+ (\sigma _2\beta _2 -(\mu _4+\mu _5y))x_2 =\kappa _y(\mu _4+\mu _5y). \end{aligned}$$

Taking *y* as free parameter we have a linear system for $x_1,x_2$, which has a unique solution so long as

9 $$\begin{aligned} \mu _1(\sigma _2\beta _2 -(\mu _4+\mu _5y))-\mu _2(\sigma _2\beta _1 -(\mu _4+\mu _5y))\ne 0. \end{aligned}$$

Observe that if both $\beta _1=\beta _2$ and $\mu _1=\mu _2$, the left-hand side of (9) is identically zero.

Assuming for simplicity $\beta _1=\beta _2=\beta ,\mu _1\ne \mu _2,\kappa _y=1$ we solve the system (7)-(8) in the parameter *y*,

$$\begin{aligned} x_1&= \tfrac{\mu _2 \mu _4 \sigma _2 - \mu _5^2 y^3 + \mu _5 [(\beta +\sigma _1)\sigma _2 - 2 \mu _4] y^2 + \left( \beta \mu _4 \sigma _2 - \mu _4^2 + \mu _2 \mu _5 \sigma _2 + \mu _4 \sigma _1 \sigma _2 - \beta \sigma _1 \sigma _2^2 \right) y }{\sigma _2 \left( \mu _1 \mu _4 - \mu _2 \mu _4 - \beta \mu _1 \sigma _2 + \beta \mu _2 \sigma _2 + \mu _1 \mu _5 y - \mu _2 \mu _5 y \right) },\\ x_2&=-\tfrac{\mu _1 \mu _4 \sigma _2 - \mu _5^2 y^3 + \mu _5 [(\beta +\sigma _1)\sigma _2 - 2 \mu _4] y^2 + \left( \beta \mu _4 \sigma _2 - \mu _4^2 + \mu _1 \mu _5 \sigma _2 + \mu _4 \sigma _1 \sigma _2 - \beta \sigma _1 \sigma _2^2 \right) y }{\sigma _2 \left( \mu _1 \mu _4 - \mu _2 \mu _4 - \beta \mu _1 \sigma _2 + \beta \mu _2 \sigma _2 + \mu _1 \mu _5 y - \mu _2 \mu _5 y \right) }. \end{aligned}$$

After substitution into (1a) we arrive to the following fifth-order polynomial in *y*

$$\begin{aligned} \Pi (y)= & {} \mu _5^3 y^5 + \mu _5^2 \left( 3 \mu _4 - 2 \beta \sigma _2 - \sigma _1 \sigma _2 \right) y^4 \\{} & {} + \, \left( 3 \mu _4^2 \mu _5 - \mu _1 \mu _5^2 \sigma _2 - \mu _2 \mu _5^2 \sigma _2 - \mu _5^2 \nu _1 \sigma _2 + \beta ^2 \mu _5 \sigma _2^2 - 4 \beta \mu _4 \mu _5 \sigma _2 \right. \\{} & {} \left. - \,2 \mu _4 \mu _5 \sigma _1 \sigma _2 + 2 \beta \mu _5 \sigma _1 \sigma _2^2 \right) y^3 \\{} & {} + \, \left( \mu _4^2 \left( \mu _4 - \sigma _1 \sigma _2 \right) - 2 \beta \mu _4^2 \sigma _2 + \beta ^2 \sigma _2^2 \left( \mu _4- \sigma _1 \sigma _2 \right) \right. \\{} & {} - \, 2 \mu _1 \mu _4 \mu _5 \sigma _2 - 2 \mu _2 \mu _4 \mu _5 \sigma _2 - 2 \mu _4 \mu _5 \nu _1 \sigma _2 \\{} & {} + \, \beta \mu _1 \mu _5 \sigma _2^2 + \beta \mu _2 \mu _5 \sigma _2^2 + \beta \mu _5 \nu _1 \sigma _2^2 + 2 \beta \mu _4 \sigma _1 \sigma _2^2 \\{} & {} + \left. \, \mu _2 \mu _5 \sigma _1 \sigma _2^2 + \mu _5 \nu _1 \sigma _1 \sigma _2^2 \right) y^2 \\{} & {} + \, \left( \beta \mu _1 \mu _4 \sigma _2^2 - \mu _2 \mu _4^2 \sigma _2 - \mu _4^2 \nu _1 \sigma _2 - \mu _1 \mu _4^2 \sigma _2 + \beta \mu _2 \mu _4 \sigma _2^2 + \beta \mu _4 \nu _1 \sigma _2^2 \right. \\{} & {} - \, \beta \mu _2 \sigma _1 \sigma _2^3 - \beta \nu _1 \sigma _1 \sigma _2^3\\{} & {} + \left. \, \mu _1 \mu _2 \mu _5 \sigma _2^2 + \mu _2 \mu _5 \nu _1 \sigma _2^2 + \mu _2 \mu _4 \sigma _1 \sigma _2^2 + \mu _4 \nu _1 \sigma _1 \sigma _2^2 \right) y \\{} & {} + \, (\mu _1 +\nu _1) \mu _2 \mu _4 \sigma _2^2 \end{aligned}$$

whose roots determine the values of *y* at equilibrium under the above conditions.

Furthermore, to ensure positivity of both $x_1,x_2$, Eq. (7) implies *y* must satisfy

$$\begin{aligned} \sigma _1 y - \frac{\mu _4y}{\sigma _2} -\frac{\mu _5y^2}{\sigma _2}>0 \end{aligned}$$

or

10 $$\begin{aligned} 0<y<\tilde{y}\equiv \frac{\sigma _1 \sigma _2-\mu _4}{\mu _5}. \end{aligned}$$

To ensure the existence of at least two positive real roots *y* of $\Pi$ as in (10) we have to impose

11 $$\begin{aligned} \Pi (0)>0,\quad \Pi (\tilde{y})>0, \quad \exists q\in (0,\tilde{y}): \Pi (q)<0. \end{aligned}$$

It holds that $\Pi (0)=(\mu _1+\nu _1) \mu _2 \mu _4 \sigma _2^{2}>0$, and

$$\begin{aligned} \Pi (\tilde{y})=\frac{\sigma _1 \sigma _2^{3}(\mu _2 \mu _5(\mu _1+\nu _1)+(\beta -\sigma _1) \mu _1(\sigma _1 \sigma _2-\mu _4))}{\mu _5} . \end{aligned}$$

For $\Pi (\tilde{y})>0$ the following inequality has to be satisfied:

$$\begin{aligned} \mu _2 \mu _5(\mu _1+\nu _1)+(\beta -\sigma _1) \mu _1(\sigma _1 \sigma _2-\mu _4)>0, \end{aligned}$$

which is equivalent to

12 $$\begin{aligned} \mu _1 \beta (\mu _4-\sigma _1 \sigma _2)<\mu _1 \sigma _1(\mu _4-\sigma _1 \sigma _2)+\mu _2 \mu _5(\mu _1+\nu _1). \end{aligned}$$

The following cases arise from (12):

1. $\mu _4-\sigma _1 \sigma _2>0$: Then the right side of (12) is positive and therefore
  $$\begin{aligned} \beta <\frac{\mu _2 \mu _5(\mu _1+\nu _1)+\mu _1 \sigma _1(\mu _4-\sigma _1 \sigma _2)}{\mu _1(\mu _4-\sigma _1 \sigma _2)}. \end{aligned}$$
2. $$\mu _4-\sigma _1 \sigma _2<0$$: Then
  - If $\mu _2 \mu _5(\mu _1+\nu _1)+\mu _1 \sigma _1(\mu _4-\sigma _1 \sigma _2)>0$, (12) is satisfied for every positive $\beta$.
  - If $$\mu _2 \mu _5(\mu _1+\nu _1)+\mu _1 \sigma _1(\mu _4-\sigma _1 \sigma _2)<0$$ then
    $$\begin{aligned} \beta >\frac{-\mu _2 \mu _5(\mu _1+\nu _1)+\mu _1 \sigma _1(\sigma _1 \sigma _2-\mu _4)}{\mu _1(\sigma _1 \sigma _2-\mu _4)}. \end{aligned}$$

If there are multiple roots of $\Pi (y)$ which satisfy the conditions for positivity, then by continuing the solution of the algebraic system of the right-hand side of (1) set to 0 for positive $\alpha \approx 0$, we expect to find some range of $\alpha >0$ where multistationarity of (1) is present. We shall give an illustration of this property in Sect. 4.

## Computational Results

Since the proposed model (1) is highly nonlinear, we shall continue the bifurcation analysis using numerical methods. We use the MatCont toolbox for numerical continuation (Dhooge et al. 2008) and Wolfram Mathematica (Wolfram Research, Inc 2023) for time integration of the system of ordinary differential equations.

Model (1) aims at casting in mathematical terms processes that occur during the initial stage of SLE. Since these predate clinical manifestations by years, not all variables in the model might be observed or measured. While some of the model parameters may be estimated in some range from experimental observations, but many are not available from experimental measurements, we shall explore the behaviour of the model by varying their values across biologically relevant ranges. We will explore several scenarios which reflect the most important dynamical features of (1).

First, we explore changes in the yield of autoantigen from NETosis $\alpha$ in Sect. 4.1. We interpret smaller values of the yield $\alpha$ as a scenario where the immune system manages to clear the neutrophil extracellular traps more efficiently, reducing the amount of exposed cytoplasmic, nuclear and granular material which could trigger an aberrant immune response.

Second, in Sect. 4.2 we also study the effect of changes in the macrophage recruitment/activation rate $\sigma _2$ on the model dynamics. Macrophage activation depends on the amount of receptors involved in pathogen binding, and recruitment and/or activation of macrophages may be influenced by biological sex and/or sex hormones.

We recall that the dysfunction of the complement system in SLE is well-known (Botto and Walport 2002; Gaipl et al. 2005). Complement protein C1q is important for an effective clearance of apoptotic material as demonstrated by experimental mouse models. C1q-deficient mice are characterised by significantly greater numbers of apoptotic bodies and autoantibody production compared with control (Botto et al. 1998); so, in our mathematical model we can suppose a positive correlation between the functionality of C1q and the rate $\nu _1$ at which late apoptotic material is produced from inappropriately cleared dying cells. Lower functionality or deficiency of C1q would be associated to a higher value of $\nu _1$. In Sect. 4.3 we explore the effect of changes of $\nu _1$ on the model dynamics.

There, we also perform bifurcation analysis using as free parameter the production rate of apoptotic material $\sigma _1$. In our model, this parameter is a generalised measure of the rate at which apoptotic cells are introduced, as a result of for example, infection, tissue damage, etc. We interpret larger values of $\sigma _1$ as a more pronounced effect of macrophages on preparing cells for apoptosis.

Finally, in Sect. 4.4 we vary the maximum pick-up rates $\beta _1,\beta _2$ to see how sensitive is the relative abundance of autoantigen $x_2$ in the total amount of antigen $x_1+x_2$.

We have chosen different sets of parameter values to illustrate the wide range of the asymptotic behaviours of model (1). Results of the numerical experiment are plotted as bifurcation diagrams, which show the values at equilibrium for the variables of model (1) as a function of a bifurcation parameter. In the bifurcation diagrams, locally asymptotically stable equilibria are plotted as a thick curve, whereas unstable equilibria are a dashed or dotted line. Solutions which are periodic in time are described in terms of the minimum and maximum value of the cycle as a function of the bifurcation parameter.

### Varying $\alpha$

For our first numerical experiment we use values

P.1 $$\begin{aligned} \begin{aligned} \mu _1&= 12, \mu _2=11, \mu _3=1.25, \mu _4=0.2; \mu _5=8.18{\times }10^{-7},\\ \sigma _1&=10^{-5},\sigma _2=9000,\sigma _3=5{\times }10^6, \nu _1=0.5, \nu _2=0.5,\\ \beta _1&=5.8{\times }10^{-4}, \beta _2=5.9{\times }10^{-4}, \beta _3=6000, \kappa _y=1, \kappa _z=10^4. \end{aligned} \end{aligned}$$

**Fig. 2:**
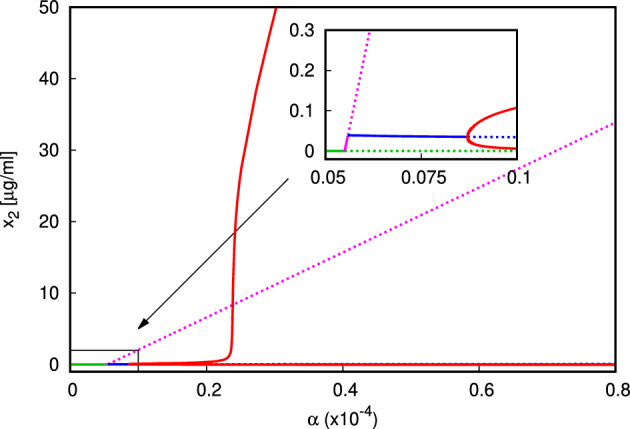
Bifurcation diagram $\alpha$ versus $x_2$ for $\alpha \in (0,8{\times }10^{-5})$. In the zoomed panel, the branch of state $E_0$ is shown in green, branch of state $E_1$ in magenta, the branch of $E_*$ in blue. The red lines mark the minima and maxima values in the limit cycle which arises from the supercritical Hopf bifurcation (parameters (P.1) (color figure online)

In Fig. 2 we present the bifurcation diagram of the values of $x_2$ as functions of $\alpha$. The values of $x_1$ are plotted in Fig. S.1 in the Supplementary Material. We vary the value of $\alpha$ in the interval $(0,2.5{\times }10^{-4})$ $\mu$g/cell. In accordance with the analytical result in Sect. 3, for small values of $\alpha$ the normal state is locally asymptotically stable. There is a transcritical bifurcation at $\alpha _0=5.5{\times }10^{-6}$ where $E_1$ appears and exchanges stability with $E_0$, which is a consequence of the analysis in Sect. 3.1. At $\alpha _1\approx 5.509{\times }10^{-6}$, $E_1$ becomes unstable and the coexistence state $E_*$ branches from the state $E_1$. This is better observed in Fig. 2; note that since in both equilibria $E_0$ and $E_1$, the value $x_1=0$, and these branching points overlap on Figure S.1 in the Supplementary Material.

The state $E_1$ is locally asymptotically stable for a very narrow range of $\alpha$. We observe the onset of supercritical Hopf bifurcation at $\alpha \approx 8.71{\times }10^{-6}$, where a stable limit cycle appears from the coexistence state $E_*$. For larger values of $\alpha$ the solution of (1a–1d) is periodic in time with growing amplitude of oscillations. For $\alpha \approx 9.15{\times }10^{-4}$ (beyond the assumed biologically relevant range of $\alpha$ in Table 2) the coexistence branch regains stability (not plotted).

For the second numerical experiment we use values

P.2 $$\begin{aligned} \begin{aligned} \mu _1&= 12,\mu _2=11,\mu _3=1.25,\mu _4=0.2,\mu _5=9{\times }10^{-6},\\ \sigma _1&=10^{-5},\sigma _2=10^5,\sigma _3=5{\times }10^6, \nu _1=0.5,\nu _2=3.33,\\ \beta _1&=\beta _2=0.002,\beta _3=1.33{\times }10^4, \kappa _y=1, \kappa _z=10^4. \end{aligned} \end{aligned}$$

**Fig. 3:**
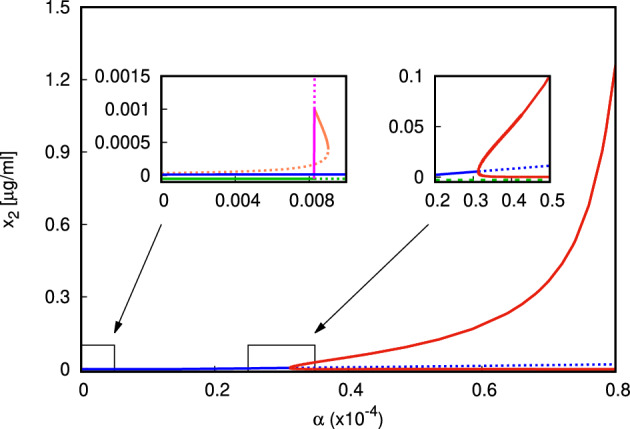
Bifurcation diagram $\alpha$ versus $x_2$ for $\alpha \in (0,8{\times }10^{-5})$, with zoomed panels for clarity of presentation (parameters given in (P.2). The branch of states $E_0$ is shown in green, the branch of $E_1$ in magenta, the two branches of type $E_*$ in blue and orange (*top left* only). The black dots represent the branching points between $E_0,E_1,E_*$, and the red dot—the supercritical Hopf bifurcation (Color figure online)

We observe the occurrence of a transcritical bifurcation from $E_0$ at $\alpha _0 \approx 8.28{\times }10^{-7}$, following the analysis in Sect. 3.1. Again, the state $E_1$ is asymptotically stable for a very narrow range of $\alpha$ (magenta branch, Fig. 3, *left zoomed panel*) before a coexistence equilibrium branches from it via a transcritical bifurcation.

The parameter set (P.2) is checked against the necessary conditions (11) for multistationarity at $\alpha =0$. Since for the selected parameters $\tilde{y} = 8.89{\times }10^4$, and further,

$$\begin{aligned} \Pi (0)=2.75{\times }10^{11}>0, \Pi (\tilde{y})=2.26{\times }10^{13}>0, \Pi \biggl (\frac{\tilde{y}}{2}\biggl )=-2.83{\times }10^{16}<0, \end{aligned}$$

the conditions (11) are satisfied and $\Pi (y)$ has at least two real roots in the interval $(0,\tilde{y})$. Furthermore $\mu _4-\sigma _1 \sigma _2=-0.8<0$ and $\mu _2 \mu _5(\mu _1+\nu _1)+\mu _1 \sigma _1(\mu _4-\sigma _1 \sigma _2)=0.11>0$, so (12) is satisfied for every positive choice of $\beta$. Due to the continuity of the solutions of the algebraic system, for $\alpha \approx 0$, there are two branches of coexistence states $E_*$ which exist for $\alpha >0$.

In Fig. 3 we present the bifurcation diagram of the equilibrium values of $x_2$ as function of $\alpha$. The values of $x_1$ are shown in Figure S.2 in the Supplementary Material. The coexistence branches are plotted in blue and orange in Fig. 3. The blue branch of states of type $E_*$ is actually disjoint from the states $E_0,E_1$ in the biologically relevant range $\alpha \ge 0$, whereas the orange branch of states of type $E_*$ bifurcates from the branch of states of type $E_1$. Numerical computation of the eigenvalues of the Jacobi matrix shows that bistability between $E_0$ and $E_*$, $E_1$ and $E_*$, or the two equilibria of coexistence type is possible in different subintervals of $\alpha \in (0,9{\times }10^{-7})$ (Fig. 3, *left zoomed panel*).

**Fig. 4:**
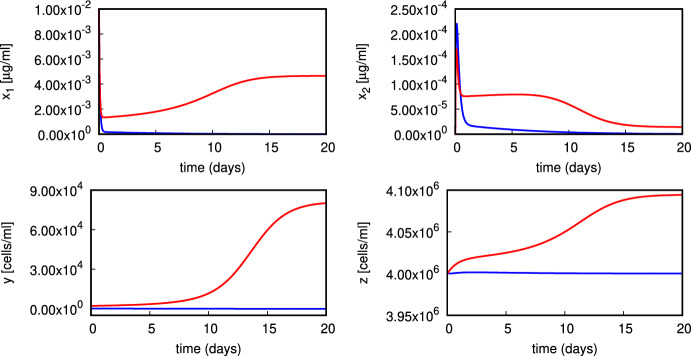
Illustration of bistability in (1) (parameters given in (P.2) with $\sigma _1=10^{-5},\sigma _2=10^5,\nu _1=0.5,\alpha =5{\times }10^{-7}$). The blue trajectory converges to the normal state $E_0$, while the red trajectory converges to the coexistence state $E_*$. Initial values are $x_1(0)=0.01, nx_2(0)= 0, y(0)= 200~\text {(blue)},2000~\text {(red)},z(0)= 4{\times }10^6$ (Color figure online)

We plot in Fig. 4 two trajectories of the system (1) to illustrate the phenomenon of bistability in (1), following the analysis of multistationarity in Sect. 3. For sufficiently small value of $\alpha$ different initial conditions lead to trajectories which converge either to the normal state $E_0$, while the red trajectory converges to the coexistence state $E_*$. Since the turnover of neutrophils *z* is rapid, the computed trajectories for *z*(*t*) converge fast towards their steady state values.

Moreover, the model (1) may exhibit coexistence of a locally asymptotically stable equilibrium and a stable limit cycle (Supplementary Material, Figure S.12 for parameter set (P.10).)

### Varying $\sigma _2$

**Fig. 5:**
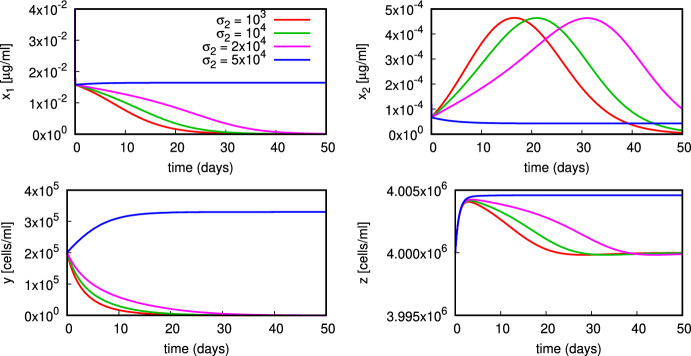
Plot of the model dynamics for different values of $\sigma _2$ (remaining parameters in (P.1) with $\alpha =5.2{\times }10^{-6}$)

**Fig. 6:**
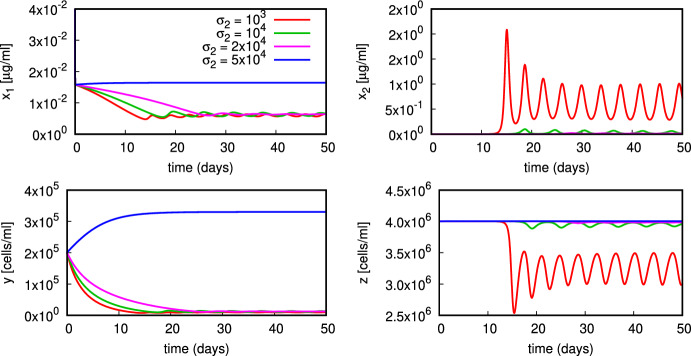
Plot of the model dynamics for different values of $\sigma _2$ (remaining parameters in (P.1) with $\alpha =6{\times }10^{-6}$)

We study the effect of the macrophage recruitment/activation rate $\sigma _2$. As mentioned previously, biological sex and/or sex hormones may influence macrophages recruitment and/or activation. This is not surprising, as sex-biased or sex hormone-dependent inflammatory responses have been previously reported  (Gaignebet et al. 2020; Gaignebet and Kararigas 2017; Horvath and Kararigas 2022; Kararigas et al. 2014; Sabbatini and Kararigas 2020a, b; Siokatas et al. 2022; Spinetti et al. 2022). Simulations of the concentrations of the two types of antigen are plotted in Figs. 5, 6 for different values of $\sigma _2$.

In Fig. 5 we observe that for increasing $\sigma _2$ the system’s asymptotic behaviour changes: from convergence to the normal state $E_0$ for $\sigma _2=10^3,10^4,2{\times }10^4$ to convergence to the coexistence state $E_*$ for $\sigma _2=5{\times }10^4$. In Fig. 5 (*bottom panels*) we plot the active macrophages’ and neutrophils’ dynamics. As $\sigma _2$ increases, so does the active macrophages’ response. This reflects on the production of apoptotic material $x_1$ via the term $\sigma _1y$ in (1a). The peak in the autoantigen $x_2$ shifts later in time if $\sigma _2$ is increased from $10^3$ to $2{\times }10^4$, but consequently the autoantigen is cleared. A similar picture emerges when $\beta _3$ is increased (Fig. S.3 in the Supplementary Material).

In the simulation in Fig. 6 with a different value of $\alpha$, we observe convergence onto a stable limit cycle for $\sigma _2=10^3$ and damped oscillations onto the coexistence state $E_*$ for $\sigma _2=10^4,2{\times }10^4,5{\times }10^4$. Note that this value of $\alpha$ is above the transcritical bifurcation value $\alpha _1$ associated to parameter set (P.1).

### Varying $\sigma _1$ and $\nu _1$

We perform numerical bifurcation analysis of the equilibria by choosing as bifurcation parameters $\sigma _1$ or $\nu _1$. Neither of them appears in the bifurcation threshold values $\alpha _0,\alpha _1$ that determine the local asymptotic stability of $E_0,E_1$ calculated in Sect. 3, and thus we must employ numerical experiment in order to analyse their effect on the appearance of equilibria branches.

**Fig. 7:**
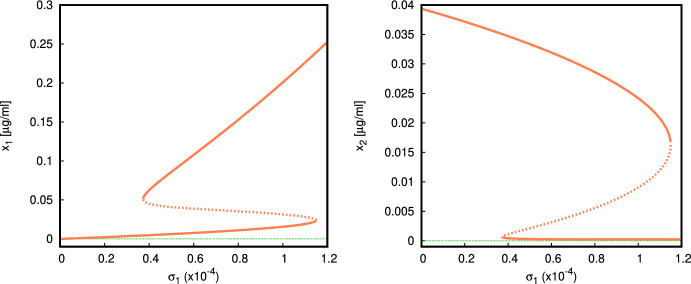
Bifurcation diagram, $\sigma _1$ versus $x_1$ (*left*), $\sigma _1$ versus $x_2$ (*right*) (parameter values given in (P.1) with $\nu _1=0.5,\sigma _2=9000,\alpha =6{\times }10^{-6}$). A range of bistability between two coexistence states $E_*$ is $\sigma _1\in (0.373{\times }10^{-4},1.15{\times }10^{-4})$

For the simulation in Fig. 7 we use parameter set (P.1) and vary the rate of production of apoptotic material $\sigma _1$ in the range $(0,1.2{\times }10^{-4})$. Observe that for the particular value of $\alpha =6{\times }10^{-6}$, analysis of the local stability of these equilibria implies that none of the stability conditions (3) for $E_0$ and (6) for $E_1$ is satisfied due to $\alpha >\alpha _1=5.509{\times }10^{-6}$. Another interesting feature is the coexistence of two locally asymptotically stable equilibria of coexistence type $E_*$ (Fig. 7). We note that as $\sigma _1$ increases, the steady state amount of autoantigen $x_2$ actually decreases. Hence, the model (1) shows that an increased rate of production of apoptotic material e.g. due to inflammatory signalling may not necessarily increase the amount of autoantigen if the immune system is already in a pathological state.

**Fig. 8:**
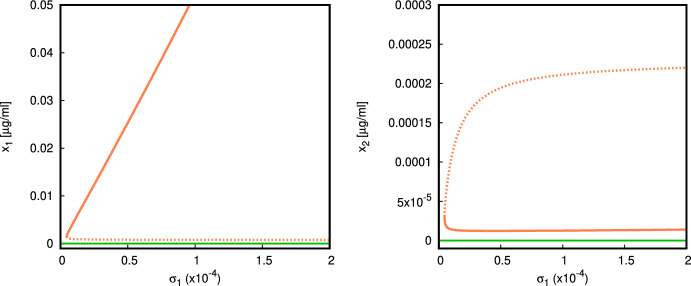
Bifurcation diagram, $\sigma _1$ versus $x_1$ (*left*), $\sigma _1$ versus $x_2$ (*right*) (parameter values given in (P.2) with $\nu _1=0.5,\sigma _2=10^5,\alpha =7{\times }10^{-7}$). There is a range of bistability between the normal $E_0$ (green) and the coexistence state $E_*$ (orange) for a range of $\sigma _1$ (Color figure online)

In Fig. 8 we use the parameter set (P.2), with $\alpha =7.5{\times }10^{-7}$, and vary $\sigma _1$. The parameter $\sigma _1$ does not enter in the local stability condition for the normal state $E_0$ derived in (3). This chosen set of parameter values provides a locally asymptotically stable steady state $E_0$ because

$$\begin{aligned} \alpha =7.5{\times }10^{-7}<\alpha _0=\frac{\mu _2 \mu _3}{\sigma _3 \nu _2}=8.25{\times }10^{-7}. \end{aligned}$$

As we increase the value of $\sigma _1$ (e.g. the rate of production of apoptotic material can increase due to an inflammatory response or tissue damage), we observe the emergence of a disconnected branch of equilibria of type $E_*$ (*coexistence*), one of which is locally asymptotically stable. The numerical bifurcation analysis presented in Fig. 8 shows that bistability between the normal state $E_0$ and the pathological coexistence state $E_*$ can appear as we vary $\sigma _1$.

**Fig. 9:**
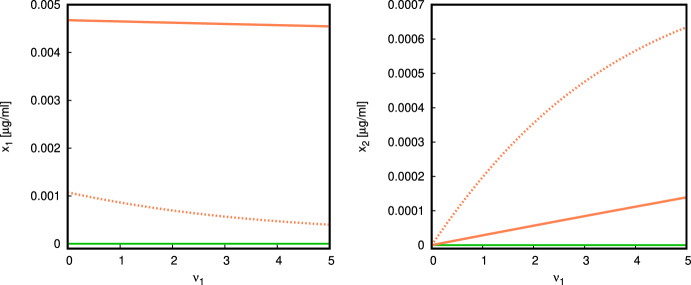
Bifurcation diagram, $\nu _1$ versus $x_1$ (*left*), $\nu _1$ versus $x_2$ (*right*) (parameter values given in (P.2) and $\sigma _1= 0.1, \sigma _2 = 10,\alpha =7.5{\times }10^{-8}$). There is bistability between the normal state $E_0$ and the coexistence $E_*$ along the whole interval $\nu _1\in (0,5]$

For the parameter $\nu _1$ the situation is similar. Bistability is possible, and occurs for an entire range of $\nu _1$ starting from 0 (Fig. 9). The parameter values are chosen so that $E_0$ is locally asymptotically stable, but at $\nu _1>0$ there exists a pair of branches of equilibria of coexistence type $E_*$, and one of them is locally asymptotically stable. Increasing $\nu _1$ leads to a saturation of the quantity of autoantigen.

### Varying $\beta _i$

In the following numerical experiments we vary the maximum pick-up rates $\beta _1,\beta _2,\beta _3$ to examine the sensitivity of the steady state values $x_1,x_2$ both in absolute and relative terms in the coexistence equilibrium. This is important as reports on macrophages in SLE patients describe a range of defects in their capacity for phagocytosis (Gaipl et al. 2005). We use the parameter values given in (P.3), while varying $\beta _1,\beta _2$.

P.3 $$\begin{aligned} \begin{aligned} \mu _1&= 10,\mu _2=11,\mu _3=1.25,\mu _4=0.2,\mu _5=8.18{\times }10^{-7},\\ \sigma _1&=10^{-5},\sigma _2=9{\times }10^3, \sigma _3=5{\times }10^6,\\ \nu _1&=0.5,\nu _2=0.05,\beta _3=600, \kappa _y=1, \kappa _z=10^4,\alpha =6{\times }10^{-5}. \end{aligned} \end{aligned}$$

Observe that for this set of parameters the steady state $E_0$ is unstable. The results are plotted as heat maps in the Supplementary Material (Fig. S.16 as absolute values for $E_*$, and as relative abundance, or percentage at equilibrium ($x_1/(x_1+x_2),x_2/(x_1+x_2)$) Fig. S.17). There is a range of $\beta _1,\beta _2$ where the coexistence state $E_*$ is unstable, so the system undergoes oscillations into a limit cycle.

Recall that for other sets of parameter values (for example, (P.2) for a range of $\alpha$, the system (1) may be bistable, with the steady state $E_0$ (no apoptotic material, no activated macrophages) being locally asymptotically stable. Results are plotted in the Supplementary Material (Fig. S.8 for varying $\beta _1$, Fig. S.9 for varying $\beta _2$, and Fig. S.11 for varying both $\beta _1,\beta _2$). Note that we do not plot those fractions that result from steady state zero values for $x_1,x_2$.

We observe that there is a slight increase of the amount of apoptotic material $x_1$ (and a decrease of the autoantigen $x_2$) in relative terms as fraction of all antigen with increasing $\beta _2$. Such behaviour is not unusual, considering the nature of the parameter $\beta _2$ as the maximum pick-up rate of autoantigen. However, the experiments show that varying $\beta _1$ and $\beta _2$ does not lead to any significant changes of the relative abundance of either type of antigen, which means that their sensitivity to those parameters is low when the yield of autoantigen formed by NETosis is low (i.e. for small values of $\alpha$).

We also use a parameter set where the yield of autoantigen from NETosis $\alpha$ is larger (Supplementary Material, (P.12)). For these values, the system has only one stable equilibrium of coexistence type. The sensitivities of the fractions to each parameter $\beta _1,\beta _2,\beta _3$ are plotted in the Supplementary Material (Fig. S.18).

We observe increasing relative abundance of autoantigen for increasing $\beta _1$ unlike the scenario plotted in Fig. S.8, which is probably due to the fact that the process of NETosis is dominant in the production of autoantigen in this case. The sensitivities of the respective fractions to $\beta _3$ are relatively low.

## Discussion and Conclusion

The proposed mathematical model is an attempt to describe some of the complex processes involved in the SLE initiation stage before the immune tolerance breaks, leading to changes in the humoral and adaptive immune response. Existing mathematical models of SLE consider the chronic stage of the disease where autoimmunity has been already established (Budu-Grajdeanu et al. 2010), focus on treatment strategies based on IL-2 for the chronic stage (Gao et al. 2022), or model the pathophysiology (kidney injury) in the case of lupus nephritis (Hao et al. 2014). Other models work with aggregate variables such as inflammatory potential and systemic inflammation (Yazdani et al. 2023) and offer limited mechanistic understanding of the processes during the disease onset.

We put forward that our model is able to capture qualitatively the most important interactions between components of the innate immune system that eventually lead to disruption of the organism homeostatis, systemic autoinflammation and the clinical manifestations of the disease. The focus of our analysis was to examine conditions leading to sustained production of autoimmunogenic material with origin either in apoptotic material, or in the process of NET formation. Being presented to T- and B-lymphocytes, such material may initiate an autoimmune response in the long-term via production of autoantibodies with broad specificity (Tsokos et al. 2016; Yaniv et al. 2015).

There are three types of steady states of the model: a *normal state* denoted by $E_0$ where no apoptotic material and autoantigen, and no activated macrophages are present; an *absence of apoptosis state* $E_1$, without activated macrophages, and material with apoptotic origin, whose only positive components are the neutrophil population and the autoantigen resulting from NETosis, and a *coexistence state* $E_*$ with positive values for all variables. The state $E_*$ is the characterised by sustained production of apoptotic material, activated macrophages and persistence of autoantigen. $E_*$ can be interpreted as a state which favours the beginning of inflammation and onset of an autoimmune response towards exposed nuclear material in blebs or NETs.

Despite its simple structure summarised in Fig. 1, our model is able to reproduce several dynamic regimes corresponding to convergence to steady states of different type, multistationarity and bistability and periodic oscillations. The condition for local asymptotic stability of the normal state $E_0$ is derived analytically in (3), and depends on the removal rate of autoantigen $\mu _2$ due to other factors, the production $\sigma _3$ and removal rate of neutrophils $\mu _3$, the rate of NET formation from encounters of neutrophils with autoantigen $\nu _2$ and the average yield $\alpha$ of autoantigen from NETosis. Therefore, suppression of the average yield of autoantigen from NETosis $\alpha$ or the NET-forming capacity of neutrophils $\nu _2$ would make the normal state locally asymptotically stable.

The bifurcation parameter we focus on initially is $\alpha$, the average yield of autoantigen as result of NETosis. As expected, for larger value of $\alpha$, the model predicts sustained production of autoantigen $x_2$—whether the system exhibits periodic oscillations or converges towards a unique stable steady state of coexistence type. Small values of $\alpha$ would be typically associated with good clearance of NETs and lower net production of autoantigen, and lower likelihood of their becoming immunogenic. However, the numerical experiments (Fig. 3 and in Supplementary Material) reveal the presence of multistationarity for small values of $\alpha$. In fact, we observe bistability for a range of $\alpha \approx 0$ between the different types of steady states: bistability is possible not only between the normal state $E_0$ and the coexistence state $E_*$, but also between $E_1$ and $E_*$, or between two states of coexistence type, or even between a coexistence state and a limit cycle (Fig. S.12 in the Supplementary Material). This means that even if the yield of autoantigen resulting from NET formation is small, due to the presence of a bistable regime, for appropriate initial conditions the system (1) can converge towards the pathological state $E_*$. In the coexistence state the presence of activated macrophages means that inflammation is sustained, whereas the sustained abundance of autoantigen may prime B- and T-lymphocytes for long-term aberrant immune responses directed towards the body itself.

The origin of the multistationarity phenomenon could lie in the mechanism we have chosen to model the pick-up of antigen by macrophages using saturated kinetics with competitive inhibition. Multistationarity is known from chemical reaction networks employing similar type of competitive inhibition for access to binding sites (Markevich et al. 2004; Wang and Sontag 2008). In fact, immune response to antigen has been modelled as a sigmoidal function in the context of cancer (Milzman et al. 2021; Zheng et al. 2008). It would be interesting to be able to explore this hypothesis using an experimental model.

Moreover, we observe that bistability between the normal state $E_0$ and the pathological coexistence state $E_*$ can appear as we increase either the rate $\sigma _1$ at which apoptotic material is introduced, or the rate $\nu _1$ at which inappropriately cleared apoptotic material in ruptured blebs becomes exposed as autoantigen to the immune system. Neither of the parameters $\nu _1,\sigma _1$ enters in the stability condition (3) for the normal state $E_0$, but the existence of bistability is revealed from numerical experiments. The maximum pick-up rates of apoptotic material and autoantigen $\beta _1,\beta _2$ affect the stability of the state $E_1$, and the onset of the coexistence state, but surprisingly, do not influence much the respective equilibrium values in $E_*$.

The bifurcation analysis presented in Fig. 8 shows that a disjoint branch of equilibria of coexistence type $E_*$ can appear for larger values of $\sigma _1$. Similarly, in Fig. 9 one stable and one unstable branch of equilibria of coexistence type $E_*$ exist for a range of $\nu _1>0$, but they do not bifurcate from the normal state branch $E_0$. Again, this property may seem counterintuitive, as low values of $\nu _1$ would be associated with efficient clearance of apoptotic material, preventing build-up of blebs that could rupture and spill immunogenic content. The presence of bistability in the model is important as it highlights a possibility where an external disturbance of the model state may tip the dynamics from one basin of attraction into another. The numerical experiment plotted in Fig. 4 shows that in a bistable regime a larger amount of activated macrophages (following for example, an environmental trigger, stress or trauma) is sufficient to tip the dynamics towards the pathological state.

Another dynamical property that our model can display is Hopf bifurcation, resulting in sustained periodic oscillations. This scenario can be interpreted as another possible path to an aberrant immune response and the onset of autoimmunity. Apoptotic waste and autoantigen are produced persistently with periods of remission where the immune system manages to partially suppress them. However, this process is not completed, leading to exhaustion of macrophage activity, accumulation of apoptotic debris and a renewed peak of autoantigen. In this case, the model predicts an innate immune response protracted in time, which may become pro-inflammatory via persistent activation of Toll-like receptors (Theofilopoulos et al. 2011; Tsokos et al. 2016) and initiate a cascade towards long-run spread of autoimmunity.

We have performed numerical experiments with different values of the recruitment or activation rate of macrophages $\sigma _2$ (Figs. 5 and 6). The rate of activation and recruitment rate of macrophages is dependent on action of hormones (Verthelyi 2001; Polan et al. 1989). As SLE is typically more common in women, a role of hormones may be hypothesised in its pathophysiology. The sex- or sex hormone-dependent recruitment or activation of macrophages can be attributed to various mechanisms. These include sex differences in the transcriptomic regulation of inflammatory genes and pathways (Ober et al. 2008). The sex steroid 17$\beta$-oestradiol (E2) also exerts a key role in immune responses, regulating pro-inflammatory cytokine expression through monocyte and macrophage regulation and affecting the expression of target genes (Kramer et al. 2007; Tiyerili et al. 2012). Interestingly, E2 has been shown to reduce lipid accumulation in female human macrophages but not in male macrophages (McCrohon et al. 1999). In particular, E2 reduced cholesteryl ester accumulation in human monocyte-derived macrophages (Corcoran et al. 2011).

Our model predicts that as $\sigma _2$ increases, the dynamics may converge onto the coexistence state with persistence of autoantigen and activated macrophages. For small $\sigma _2$, however, the system may also present a stable limit cycle. If macrophages are insufficiently activated, then the clearance of apoptotic material is impaired leading to accumulation of exposed nuclear contents such as chromatin which can initiate an autoimmune response. For larger values of $\sigma _2$, there are damped oscillations onto a steady state of low quantity of autoantigen (Fig. 6). Thus, our model, while in general predicting a persistent production of autoantigen and persistent activation of macrophages for larger values of $\sigma _2$, does not include explicitly transcriptomic or signalling mechanisms, thus not excluding different routes that lead to initiation of autoimmunity.

Both locally asymptotically stable states of coexistence type $E_*$ and limit cycles resulting from a Hopf bifurcation correspond to a pathological, aberrant immune response. Persistence or periodic cycling of autoantigen could have implications in the further development of a long-term autoimmune reaction, in particular the production of autoantibodies directed against the complement protein C1q. As C1q binds to exposed nuclear material from blebs, it forms a complex, and the persistence of antigen presentation to B-lymphocytes causes the production of antibodies against C1q – a widely accepted hallmark of lupus (Schaller et al. 2009; Tsokos 2020). As autoantibodies in SLE are result of autoantigen stimulation (Schaller et al. 2009), we would argue that the proposed model represents fairly well the mechanisms at work during the initiation stage of the disease.

Our model also has limitations which we briefly describe here. The first and foremost is that we focus only on one type of APCs (macrophages) because they are generally responsible in clearing apoptotic cells, and their response to exposed nuclear material is pro-inflammatory (Marée et al. 2006). The macrophage population we model under the variable *y* represents a generic population of macrophages which are recruited and activated in tissue to clear the dying cell material on the one hand, and the by-products which could be immunogenic, including exposed self from NETs or apoptotic blebs collect the two types of antigen. The second limitation is that we do not include dendritic cells (DCs) explicitly in the model for two reasons: first, in order to keep its structure simpler and, second, because we are not modelling activation of naïve T-lymphocytes and the initialisation cascade of the adaptive immune response via antigen presentation to B-lymphocytes in the lymph node. DCs have been shown to be activated by the contents of apoptotic blebs (Dieker et al. 2015), and NETs (Tsokos 2020). In particular, plasmacytoid DCs are powerful producers of type-I interferon, a pro-inflammatory cytokine with effect on broad range of immune cells, so DCs are candidate cells for inclusion in a future extension of the model. While currently it is known that multiple cell types are capable of NETosis (Vorobjeva and Chernyak 2020), in model (1) the cells which perform it are neutrophils because they are the most common type of leukocytes. Finally, for the sake of simplicity of the model equations and, the signalling feedback between APCs and neutrophils is modelled implicitly. If further variables are added to the model, other dynamical regimes could be possible (e.g. chaos). However, we leave this for future work.

NETosis is suspected to be a key factor in the initiation of the an aberrant immune response observed in experimental models of lupus (Dieker et al. 2016; Villanueva et al. 2011). Our model demonstrates that increased yield of autoantigen production from NETosis is a sufficient condition for the establishment and maintenance of apoptotic waste and autoantigen production. However, there are cases where the production of autoantigen can persist over time in a convergent or oscillatory manner despite a weak yield of nuclear material from NETs. This is due to the property of bistability in the model, where the healthy normal state and the pathological disease state coexist side by side as locally asymptotically stable equilibria.

### Supplementary Information

Below is the link to the electronic supplementary material.

Supplementary file 1 (pdf 702 KB)

## Data Availability

The software code and the simulation results that support the findings of this study are available from the corresponding author on reasonable request.
