# Supplementary material for: Modelling the Impact of NETosis During the Initial Stage of Systemic Lupus Erythematosus
Source: Bull Math Biol. 2024 Apr 28;86(6):66. doi: 10.1007/s11538-024-01291-3 (PMC11056343; doi:10.1007/s11538-024-01291-3)
Supplement: Supplementary file 1 — (pdf 702 KB) [file 11538_2024_1291_MOESM1_ESM.pdf]

Supplementary Material to *Modelling the Impact of NETosis During the Initial Stage of Systemic Lupus Erythematosus*

Vladimira Suvandjieva, Ivanka Tsacheva, Marlene Santos, Georgios Kararigas, and Peter Rashkov

1 Supplementary Tables

Parameter	Definition	Value/Range	Unit	Reference
α	Yield of autoantigen from NETosis	$[0, 2.5\times10^{-4}]$	μg/cell	estimated
β_1	Maximum pick-up rate of apoptotic matter by macrophages	0.002 $[0.5\times10^{-4}, 4.05\times10^{-3}]$	μg/(cell day)	estimated
β_2	Maximum pick-up rate of autoantigen by macrophages	0.002 $[0.5\times10^{-4}, 4.05\times10^{-3}]$	μg/(cell day)	estimated
β_3	Maximum activation rate of neutrophils by macrophages	as indicated	cells/(ml day)	estimated
κ_y	Michaelis constant, pick-up rate of antigen by macrophages	1	μg/ml	guess
κ_z	Michaelis constant, activation rate of neutrophils by macrophages	10^4	cells/ml	guess
μ_1	Removal rate of apoptotic matter	$[10, 12]$	day^{-1}	estimated
μ_2	Removal rate of autoantigen	$[11, 12]$	day^{-1}	estimated
μ_3	Removal rate of neutrophils	$[0.86, 1.28]$	day^{-1}	[4]
μ_4	Removal rate of macrophages	0.2	day^{-1}	[1, 4]
μ_5	Crowding coefficient, macrophages	as indicated	(cells day)$^{-1}$	estimated
ν_1	Production rate of late apoptotic matter (blebs)	$0.05, 0.5$	day^{-1}	[3]
ν_2	NETosis rate	as indicated	(μg/ml day)$^{-1}$	guess
σ_1	Production rate of apoptotic matter	10^{-5}	μg/(cell day)	guess
σ_2	Activation/recruitment rate of macrophages	as indicated	cells/μg	estimated
σ_3	Production of neutrophils	$[3.7\times10^6, 5\times10^6]$	cells/(ml day)	[4, 6]

Table S.1: Parameter definitions and values used in the numerical experiments.

Parameter values used in the numerical experiments in the main text:

2 Estimates for the analytical solution

The estimates for non-negativity and boundedness of the solutions of the model are made for times $t > 0$. The model equations are

$$x_1' = \sigma_1 y - \frac{\beta_1 y x_1}{\kappa_y + x_1 + x_2} - \nu_1 x_1 - \mu_1 x_1 \tag{1a}$$

$$x_2' = \nu_1 x_1 - \frac{\beta_2 y x_2}{\kappa_y + x_1 + x_2} + \alpha \nu_2 z x_2 - \mu_2 x_2 \tag{1b}$$

$$y' = \left(\frac{\beta_1 y x_1}{\kappa_y + x_1 + x_2} + \frac{\beta_2 y x_2}{\kappa_y + x_1 + x_2} \right) \sigma_2 - \mu_4 y - \mu_5 y^2 \tag{1c}$$

$$z' = \sigma_3 + \frac{\beta_3 y}{\kappa_z + y} - \mu_3 z - \nu_2 z x_2 \tag{1d}$$

Proposition 1 *The solutions to* (1) *remain non-negative in time* $t > 0$ *for non-negative initial values* $x_1(0), x_2(0), y(0), z(0)$.

Proof. Since the right-hand-side of (1) is continuous and continuously differentiable, the initial value problem has a unique solution for some positive time. We have that $x_1(0) \geq 0, x_2(0) \geq 0, y(0) \geq 0, z(0) \geq 0$.

Assume that the solution of (1) eventually leaves the non-negative orthant of $\mathbb{R}^4$ and take $\tau := \inf\{t > 0 : x_1(t) < 0, x_2(t) < 0, y(t) < 0, \text{ or } z(t) < 0\}$. Note that $\tau < \infty$.

Consider equation (1c) and observe that for $t \leq \tau$,

$$y' \geq -(\mu_4 + \mu_5 y) y.$$

For $\tilde{y}(\tau) = 0$ the equation

$$\tilde{y}' = -(\mu_4 + \mu_5 \tilde{y}) \tilde{y} \tag{2}$$

has a solution $\tilde{y}(t) \equiv 0, t \geq \tau$. The uniqueness theorem for solutions to ODEs implies that for initial condition $\tilde{y}(\tau) > 0$, the solution to (2) cannot take the value 0 on $t > \tau$, and by continuity $\tilde{y}(t) > 0$ for $t > \tau$. Thus, by setting $\tilde{y}(\tau) = y(\tau)$ and applying the comparison theorem for ODEs, we have $y(t) \geq \tilde{y}(t) > 0, t > \tau$, unless $y \equiv 0$.

Consider equation (1d). We have for $t \leq \tau$

$$z' > -\mu_3 z - \nu_2 x_2 z, \tag{3}$$

so there exists $\varepsilon_4 > 0$ such that

$$z(t) > z(\tau) \exp \left(- \int_\tau^t (\mu_3 + \nu_2 x_2(s)) ds \right) \geq 0, \quad t \in (\tau, \tau + \varepsilon_4).$$

Now take equation (1a) and observe that for $t \leq \tau$

$$x_1' \geq -\left(\frac{\beta_1}{\kappa_y} y + \nu_1 + \mu_1\right) x_1,$$

so there exists $\varepsilon_1 > 0$ such that

$$x_1(t) \geq x_1(\tau) \exp\left(-\int_\tau^t \left(\frac{\beta_1}{\kappa_y} y(s) + \nu_1 + \mu_1\right) ds\right) \geq 0, \quad t \in (\tau, \tau + \varepsilon_1).$$

Equation (1b) can be treated similarly to obtain that there exists $\varepsilon_2 > 0$ such that

$$x_2(t) \geq x_2(\tau) \exp\left(-\int_\tau^t \left(\frac{\beta_2}{\kappa_y} y(s) + \mu_2\right) ds\right) \geq 0, \quad t \in (\tau, \tau + \varepsilon_2).$$

These inequalities show that the solution $(x_1(t), x_2(t), y(t), z(t))$ of (1) remains non-negative for $t \in [\tau, \tau + \min_{i=1,2,4} \varepsilon_i)$, which is a contradiction to the initial assumption on τ.

□

Proposition 2 *The solutions to* (1) *remain bounded in time* $t > 0$ *for non-negative initial values* $x_1(0), x_2(0), y(0), z(0)$.

Proof. Recall that $A = (\beta_1 + \beta_2)\sigma_2 - \mu_4$ is assumed to be strictly positive in order to prevent $\lim_{t\to\infty} y(t) = 0$ for all choices of $y(0) > 0$.

From (1c) we get

$$\begin{aligned} y'(t) &\leq Ay(t) - \mu_5 y(t)^2 = 2Ay(t) - \mu_5 y(t)^2 - Ay(t) \leq \\ &\leq \frac{A^2}{\mu_5} - Ay(t), \end{aligned}$$

Thus, Gronwall's inequality bring us to the estimate

$$\begin{aligned} y(t) &\leq y(0) \exp(-\int_0^t A ds) + \int_0^t \frac{A^2}{\mu_5} e^{-At+As} ds \\ &= y(0)e^{-At} + \frac{A^2}{\mu_5} e^{-At} \frac{e^{At} - 1}{A} \\ &= y(0)e^{-At} + \frac{A}{\mu_5}(1 - e^{-At}) \\ &\leq \max\left\{y(0), \frac{A}{\mu_5}\right\}. \end{aligned} \tag{4}$$

From (1a) and the estimate (4) we have

$$x_1'(t) \le \sigma_1 y(t) - (\nu_1+\mu_1)x_1(t) \le B - (\nu_1+\mu_1)x_1(t),$$

where $B = \sigma_1 \max\{y(0), \frac{A}{\mu_5}\}$.

Then Gronwall's inequality leads to:

$$\begin{aligned}
x_1(t) &\le x_1(0)\exp(-\int_0^t (\nu_1+\mu_1)ds) + \int_0^t Be^{-(\nu_1+\mu_1)t+(\nu_1+\mu_1)s}ds \\
&= x_1(0)e^{-(\nu_1+\mu_1)t} + Be^{-(\nu_1+\mu_1)t}\frac{e^{(\nu_1+\mu_1)t-1}}{\nu_1+\mu_1} \\
&= x_1(0)e^{-(\nu_1+\mu_1)t} + B(1-e^{-(\nu_1+\mu_1)t}) \\
&\le \max\{x_1(0), B\}.
\end{aligned} \tag{5}$$

From (1d) we have

$$z'(t) \le \sigma_3 + \beta_3 - \mu_3 z(t).$$

Then the following estimate holds:

$$\begin{aligned}
z(t) &\le z(0)e^{-\mu_3 t} + \int_0^t (\beta_3+\sigma_3)e^{-\mu_3 t+\mu_3 s}ds \\
&= e^{-\mu_3 t}\left(z(0) - \frac{\beta_3+\sigma_3}{\mu_3}\right) + \frac{\beta_3+\sigma_3}{\mu_3} \\
&\le \max\left\{z(0), \frac{\beta_3+\sigma_3}{\mu_3}\right\}.
\end{aligned} \tag{6}$$

Last, we denote $w(t) = x_1(t) + x_2(t) + \alpha z(t)$.

$$\begin{aligned}
w'(t) &\le \sigma_1 y(t) - \mu_1 x_1(t) - \mu_2 x_2(t) - \mu_3 \alpha z(t) + \alpha\sigma_3 + \alpha\frac{\beta_3 y(t)}{\kappa_z + y(t)} \\
&\le C - \mu_1 x_1(t) - \mu_2 x_2(t) - \alpha\mu_3 z(t) \le C - \tilde{\mu} w(t),
\end{aligned} \tag{7}$$

where $\tilde{\mu} := \min\{\mu_1, \mu_2, \mu_3\}$.

Proposition 1 implies $w(t) \ge 0$, for all $t \ge 0$. Next, Gronwall's inequality gives

$$\begin{aligned}
w(t) &\le w(0)\exp(-\tilde{\mu}t) + C\int_0^t e^{-\tilde{\mu}t+\tilde{\mu}s}ds \\
&= e^{-\tilde{\mu}t}\left(w(0) - \frac{C}{\tilde{\mu}}\right) + \frac{C}{\tilde{\mu}}.
\end{aligned} \tag{8}$$

The boundedness of $x_2(t)$ follows from (5), (6), (8) and the non-negativity of $w(t)$. □

3 Parameter estimation

Antigen: The value range for the yield of autoantigen from NETosis α is chosen between 0 and 25% of a single cell mass, or up to 0.25 ng/cell, if the mass of a neutrophil cell is taken as 1 ng (10^{-9} g), in line with the estimate of cell mass made in [2].

The maximum pick-up rates of antigen β_1, β_2 are taken to lie within the range $[0.05, 4.05]$ ng/(cell day) or $[0.5\times10^{-4}, 4.05\times10^{-3}]$ μg/(cell day).

The value $\nu_1 = 0.5$ corresponds to the observation from *in vitro* measurement of the time for leakage of nucleosome content from apoptotic Jurkat cells [3]. This study shows that apoptotic cells transition to necrotic matter by release of nucleosomes in 24-48 hours (hence the rate $\nu_1 = 0.5$/day). However, since this estimate comes from an *in vitro* assay, we also take a 10-fold lower value ($\nu_1 = 0.05$/day) and vary ν_1 as a bifurcation parameter to account for the action of any additional innate immune mechanisms that we do not model explicitly. In accordance with this estimate we set the removal of apoptotic matter and necrotic matter to occur on a much faster scale of 2-$2\frac{1}{2}$ hours leading to the estimates of rates $\mu_1, \mu_2 \in [10, 12]$/day.

Macrophages: The removal rate of macrophages $\mu_4 = 0.2$ corresponds to a life-span of 5 days, comparable to the estimate provided in [4] (life-span of 4-7 days for blood monocytes). The values for $\beta_1, \beta_2, \sigma_2, \mu_5$ are chosen to correspond to a maximum density of recruited macrophages in the order of $10^6 - 10^7$ cells/ml, similar to the estimates of [1].

Neutrophils: The removal rate of neutrophils μ_3 is computed according to the range of their half-life reported by [4] (half-life of 13-19 hours), so $\mu_3 = 1.25 \in [\frac{24\ln 2}{19}, \frac{24\ln 2}{13}] = [0.86, 1.28]$ and this value is also close to the range used in [5].

The parameter σ_3 is chosen to bring the steady state value of $z = \sigma_3/\mu_3$ for the normal state E_0 within the range of neutrophil count for healthy volunteers 3×10^6 to 10^7 cells/ml [6]. We let accordingly σ_3 take values in the interval $(3.7\times10^6, 5\times10^6)$.

4 Supplementary Figures

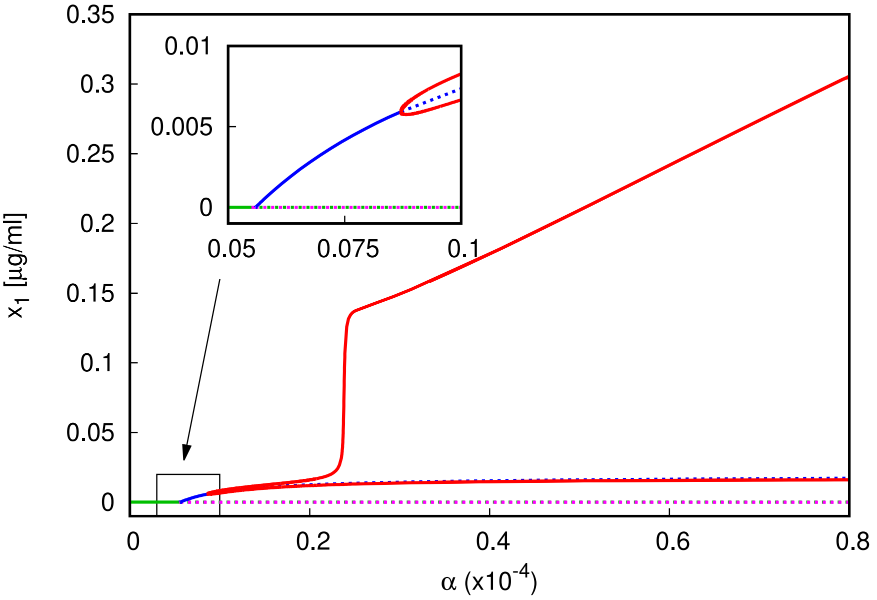

Figure S.1: Bifurcation diagram α vs. x_1 for $\alpha \in (0, 8{\times}10^{-5})$. In the zoomed panel, the branch of state E_0 is shown in green, branch of state E_1 in magenta, the branch of E_* in blue. The red lines mark the minima and maxima values in the limit cycle which arises from the supercritical Hopf bifurcation (parameters (P.1) in the main text).

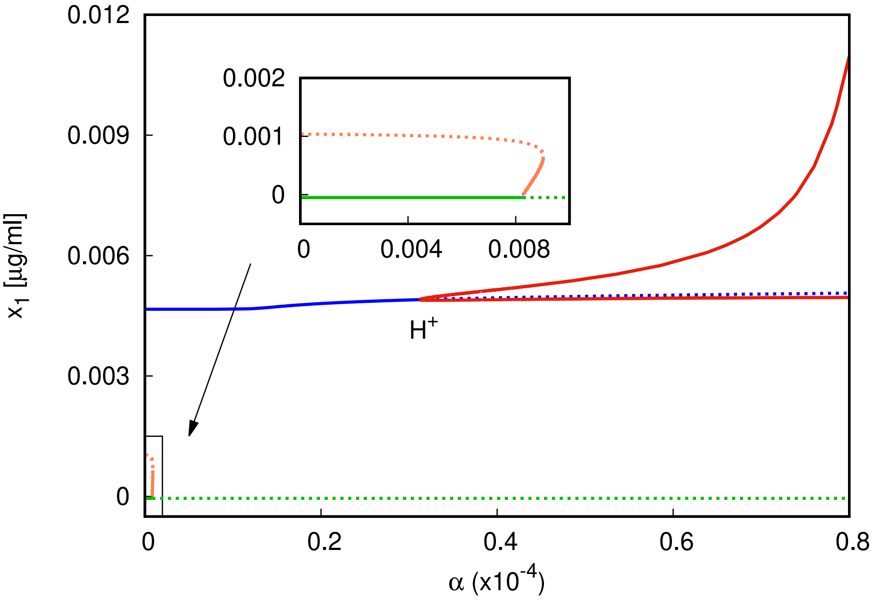

Figure S.2: Bifurcation diagram α vs. x_1 for $\alpha \in (0, 8\times 10^{-5})$, with zoomed panels for clarity of presentation (parameters (P.2) in the main text). The branch of states E_0 is shown in green, and the two branches of type E_* in blue and orange (*top*). The branch of E_1 is not plotted as it overlaps with E_0. H^+ is the start of the supercritical Hopf bifurcation.

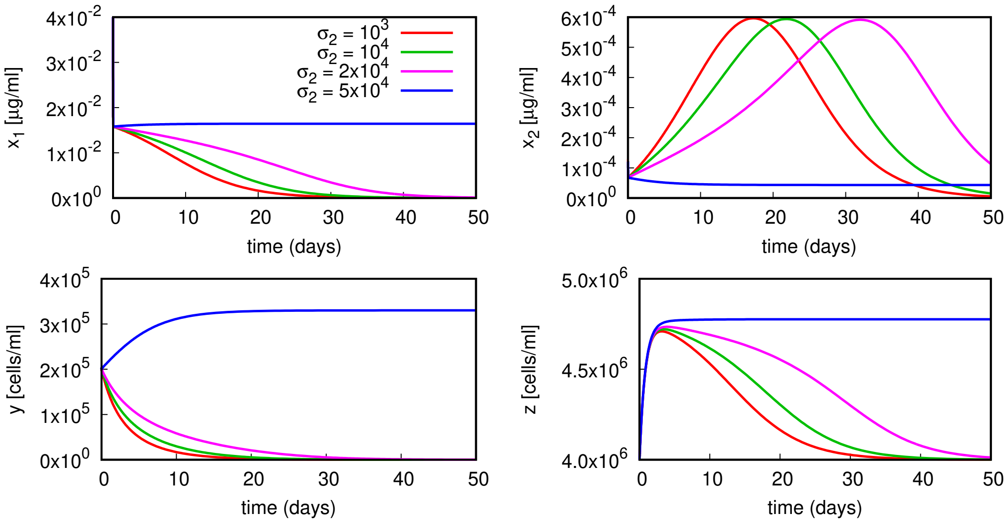

Figure S.3: Plot of the model dynamics for different values of σ_2 (remaining parameters in (P.1) with $\alpha = 5.2\times 10^{-6}, \beta = 10^6$).

4.1 Additional parameter sets

We also perform simulations for modified rates of NETosis and neutrophil dynamics using the following parameter set:

$$\begin{aligned}
&\mu_1 = 12, \mu_2 = 11, \mu_3 = 0.93, \mu_4 = 0.2, \mu_5 = 9\times10^{-6},\\
&\sigma_1 = 10^{-5}, \sigma_2 = 10^5, \sigma_3 = 3.7\times10^6,\\
&\nu_1 = 0.5, \nu_2 = 0.37,\\
&\beta_1 = 2\times10^{-3}, \beta_2 = 2\times10^{-3}, \beta_3 = 1.33\times10^4,\\
&\kappa_y = 1, \kappa_z = 10^4.
\end{aligned} \tag{P.9}$$

These values meet the conditions for multistationarity of the model (1) for $\alpha = 0$ described in the main text, and the existence of bistability in the vicinity of $\alpha = 0$ is verified numerically.

Figures S.4 and S.5 show the bifurcation structure within the biologically relevant range of α. A supercritical Hopf bifurcation H^+ on the blue coexistence branch at $\alpha = 2.91\times10^{-4}$ leads to the emergence of a stable limit cycle. We note that for much larger values of α in the interval $[0.0122, 0.0125]$ we observe multistationarity on the blue branch, with three coexistence-type states which exist in parallel and all of them being unstable for $\alpha \in [0.0122, 0.0123]$ (*not shown*). However, this interval is outside the biologically assumed range for α.

We also explore the effect of varying the rates σ_1 (Figure S.6) and ν_1 (Figure S.7).

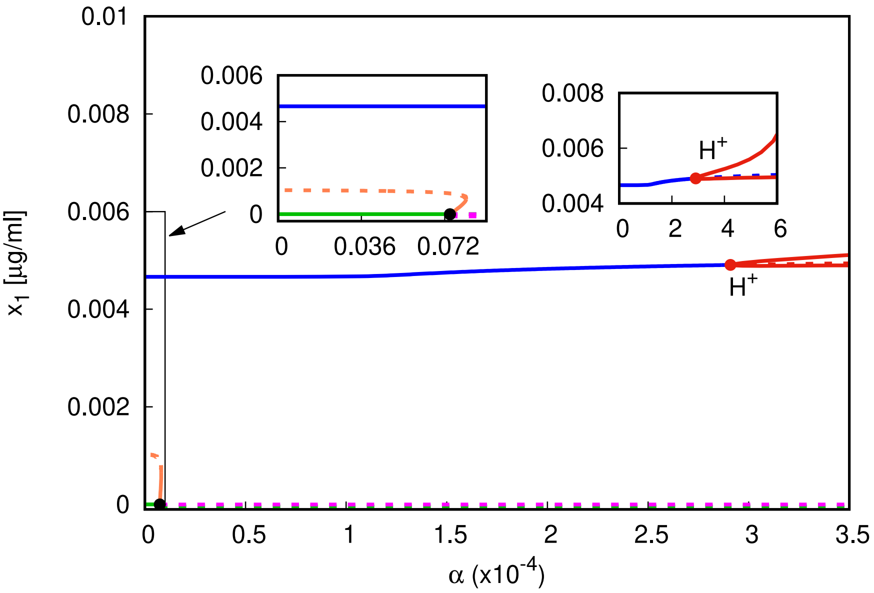

Figure S.4: Bifurcation diagram α vs. x_1, for parameter values given in (P.9) and $\alpha \in (0, 3.5{\times}10^{-4})$. Zoomed panels for $\alpha \in (0, 8{\times}10^{-6})$ and $(0, 6{\times}10^{-4})$ show the bifurcation structure in more detail. The branch of states E_0 is shown in green, the branch of E_1 in magenta, the two branches of type E_* in blue and orange. The black dots represent the branching points between E_0, E_1, E_*, and the red dot - the supercritical Hopf bifurcation.

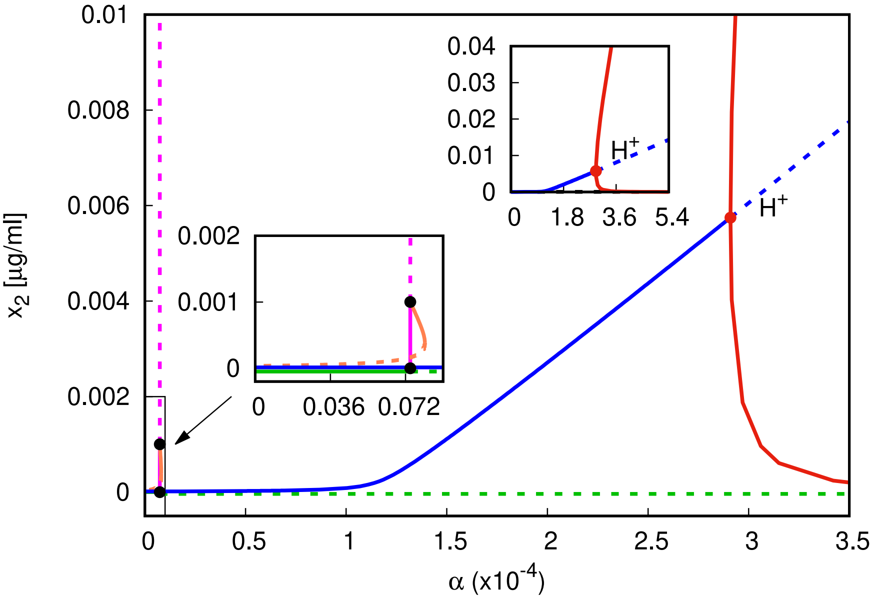

Figure S.5: Bifurcation diagram α vs. x_2, for parameter values given in (P.9) and $\alpha \in (0, 3.5\times10^{-4})$. Zoomed panels for $\alpha \in (0, 8\times10^{-6})$ and $(0, 5.4\times10^{-4})$ show the bifurcation structure in more detail. The branch of states E_0 is shown in green, the branch of E_1 in magenta, the two branches of type E_* in blue and orange (*top left* only). The black dots represent the branching points between E_0, E_1, E_*, and the red dot - the supercritical Hopf bifurcation.

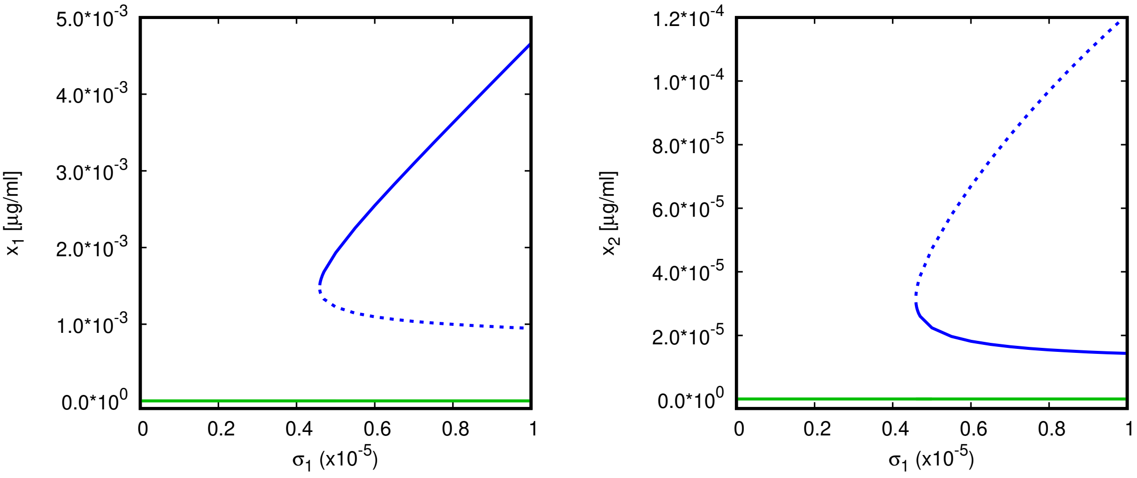

Figure S.6: Bifurcation diagram, σ_1 vs. x_1 (*left*), σ_1 vs. x_2 (*right*) (parameter values given in (P.9) and $\alpha = 6.75\times10^{-6}$). There is a range of bistability between the normal E_0 (green) and the coexistence state E_* (blue) for a range of σ_1.

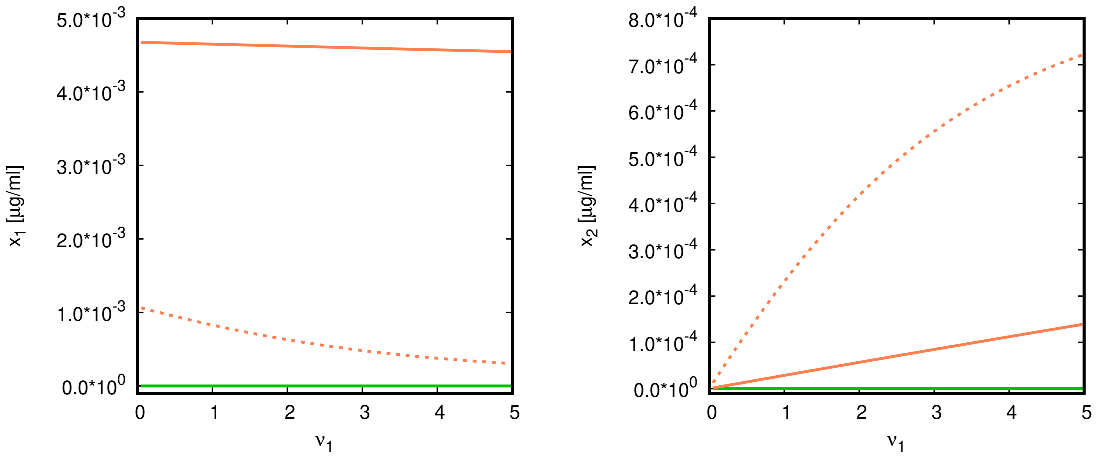

Figure S.7: Bifurcation diagram, ν_1 vs. x_1 (*left*), ν_1 vs. x_2 (*right*) (parameter values given in (P.9) and $\alpha = 6.75{\times}10^{-6}$). There is bistability between the normal state E_0 and the coexistence E_* along the whole interval $\nu_1 \in (0, 5]$.

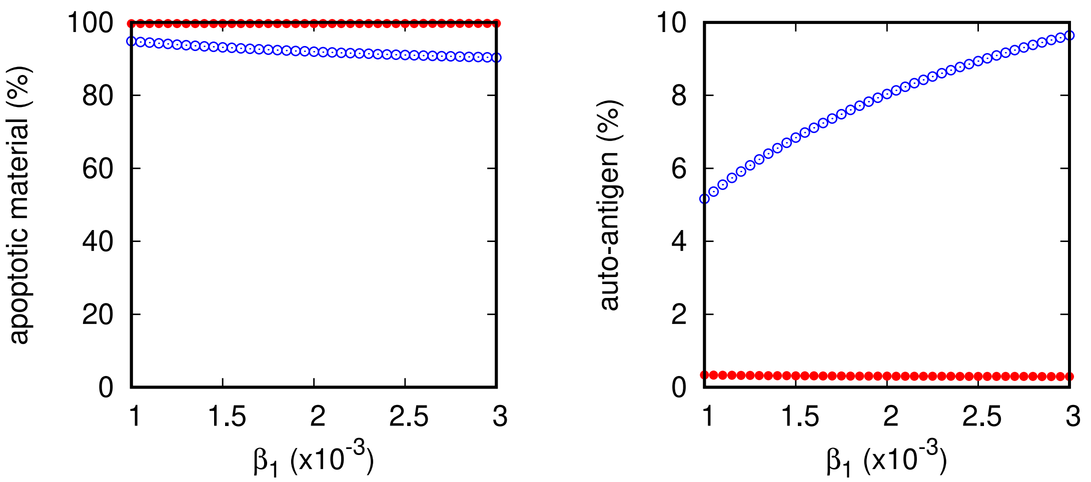

Figure S.8: Fractions of the two types of antigen as function of β_1. Red solid dot represents the fraction at the locally stable equilibrium E_*, blue circle at the unstable equilibrium E_*. Parameter values given in (P.9) with $\beta_2 = 2{\times}10^{-3}, \alpha = 5.62{\times}10^{-6}$.

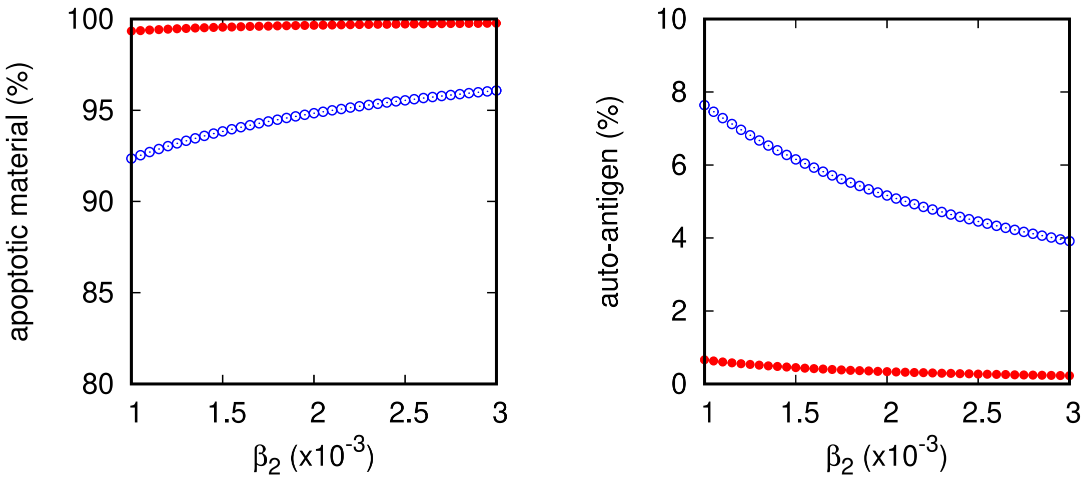

Figure S.9: Fractions of the two types of antigen as function of β_2. Red solid dot represents the fraction at the locally stable equilibrium E_*, blue circle at the unstable equilibrium E_*. Parameter values given in (P.9) with $\beta_1 = 2\times10^{-3}, \alpha = 5.62\times10^{-6}$.

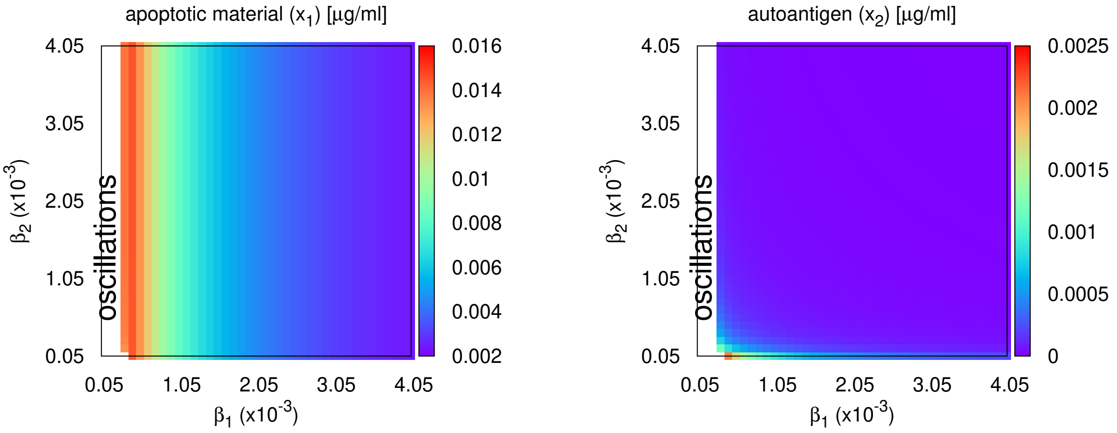

Figure S.10: Steady state values of the two types of antigen as function of β_1, β_2. Remaining parameter values given in (P.9) with $\alpha = 6.75\times10^{-6}$.

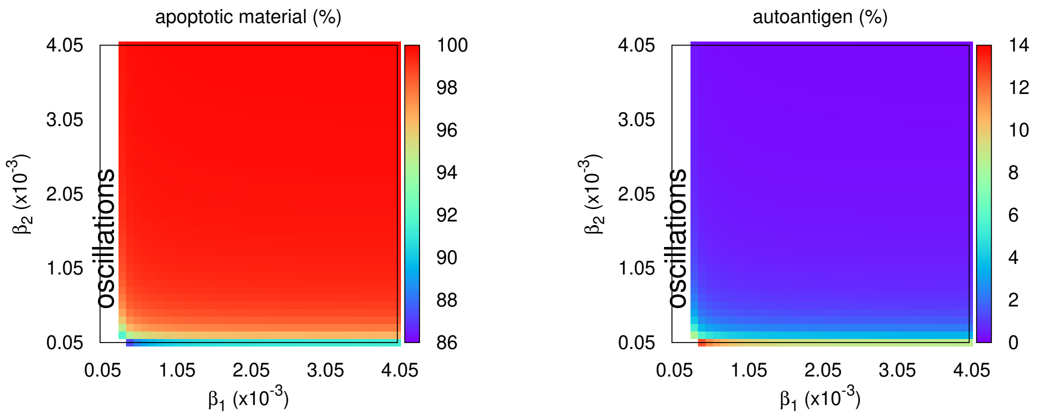

Figure S.11: Fractions of the two types of antigen as function of β_1, β_2. Remaining parameter values given in (P.9) with $\alpha = 6.75{\times}10^{-6}$.

The parameter set P.10 is characterised by a lower value for ν_1:

$$\begin{aligned}
&\mu_1 = 12, \mu_2 = 11, \mu_3 = 1.25, \mu_4 = 0.2, \mu_5 = 9\times10^{-6},\\
&\sigma_1 = 10^{-5}, \sigma_2 = 10^5, \sigma_3 = 5\times10^6,\\
&\beta_1 = 2\times10^{-3}, \beta_2 = 2\times10^{-3}, \beta_3 = 1.33\times10^5\\
&\kappa_y = 1, \kappa_z = 10^4, \nu_1 = 0.05, \nu_2 = 3.33.
\end{aligned} \tag{P.10}$$

The numerical bifurcation analysis presented in Figures S.12 and S.13 reveals a regime where a stable limit cycle coexists with a steady state of type E_* (blue branch).

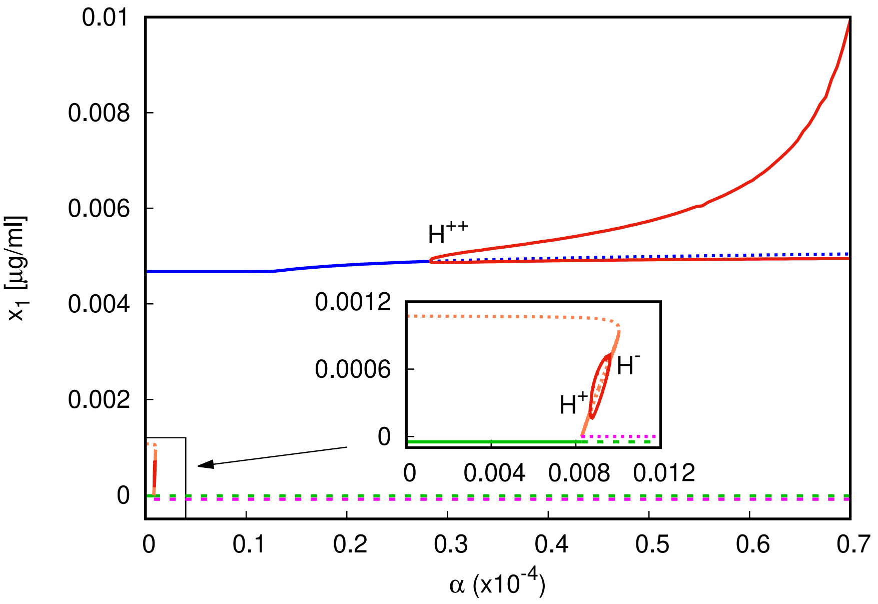

Figure S.12: Bifurcation diagram, α vs. x_1 for $\alpha \in (0, 1.2\times10^{-4})$. The branch of state E_0 is plotted in green, branch of E_* in blue. The red lines mark the minima and maxima values in the limit cycle which arises from the supercritical Hopf bifurcations at H^-, H^+ (parameter values in (P.10)).

Another supercritical Hopf bifurcation H^{++} makes the blue coexistence branch lose stability at $\alpha \approx 0.282\times10^{-4}$. The oscillations inside the limit cycle gradually increase in amplitude.

If we keep the parameters (P.10) but reduce ν_2 to $\nu_2 = 0.033$, we obtain similar dynamics, except that the transcritical bifurcations are shifted for larger values of α. The numerical bifurcation analysis presented in Figures S.14 and S.15 reveals a regime where a stable limit cycle again coexists with a steady state of type E_* (blue branch). The blue coexistence-type branch E_* is locally asymptotically stable over the entire biologically relevant range of α before a supercritical Hopf bifurcation occurs at $\alpha = 2.8195\times10^{-3}$

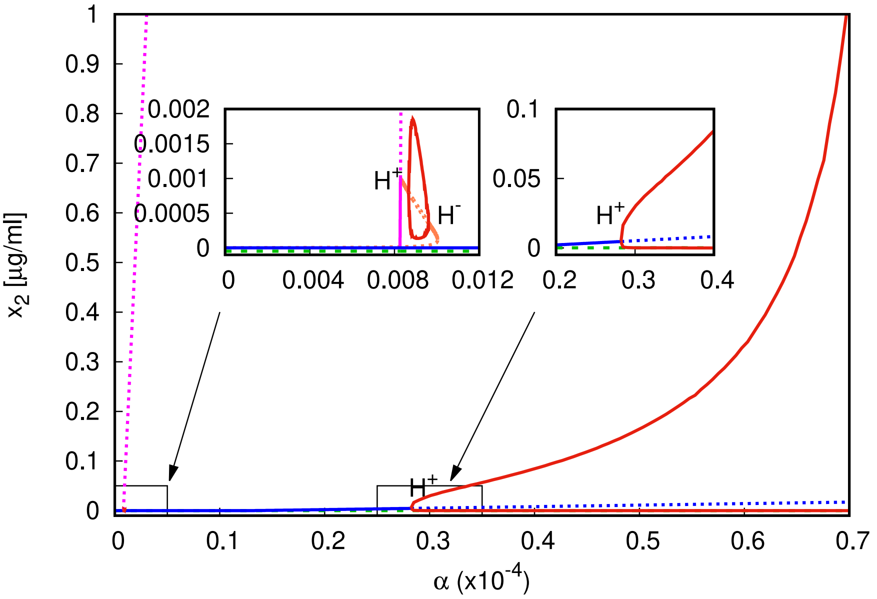

Figure S.13: Bifurcation diagram, α vs. x_2 for $\alpha \in (0, 1.2{\times}10^{-4})$. The branch of state E_0 is shown in green, branch of E_1 in magenta, and branch of E_* in blue. Zoomed panel for $\alpha \in (0, 10^{-5})$. The red lines mark the minima and maxima values in the limit cycle which arises from the supercritical Hopf bifurcations at H^-, H^+ (parameter values in (P.10)).

(*not shown*).

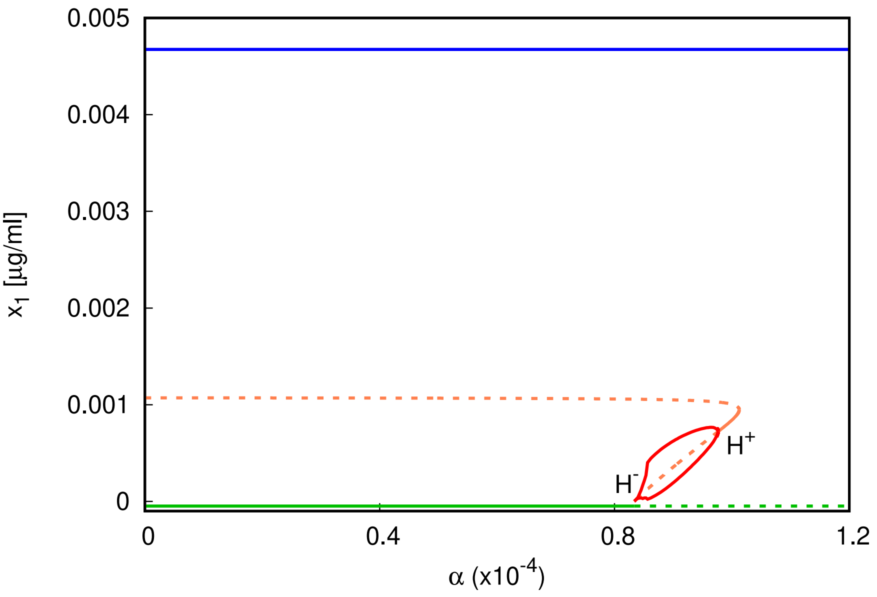

Figure S.14: Bifurcation diagram, α vs. x_1 for $\alpha \in (0, 1.2{\times}10^{-4})$. The branch of state E_0 is plotted in green, branch of E_* in blue. The red lines mark the minima and maxima values in the limit cycle which arises from the supercritical Hopf bifurcations at H^-, H^+ (parameter values in (P.10), but $\nu_2 = 0.033$).

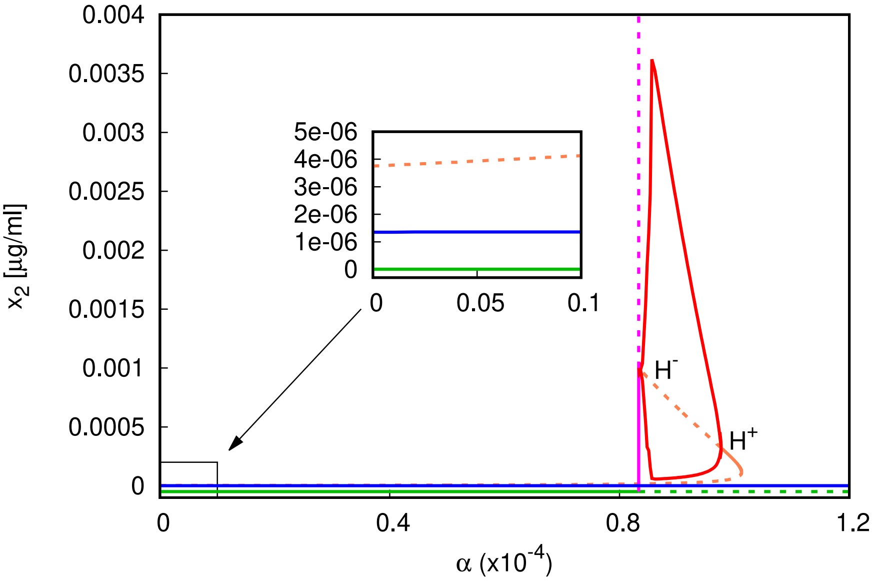

Figure S.15: Bifurcation diagram, α vs. x_2 for $\alpha \in (0, 1.2{\times}10^{-4})$. The branch of state E_0 is shown in green, branch of E_1 in magenta, and branch of E_* in blue. Zoomed panel for $\alpha \in (0, 10^{-5})$. The red lines mark the minima and maxima values in the limit cycle which arises from the supercritical Hopf bifurcations at H^-, H^+ (parameter values in (P.10) but $\nu_2 = 0.033$).

Sensitivity of the fractions of the antigen to variation of β_1, β_2 is done with the following parameter set:

$$\begin{aligned} &\mu_1 = 10, \mu_2 = 11, \mu_3 = 1.25, \mu_4 = 0.2, \mu_5 = 8.18\times 10^{-7}, \\ &\sigma_1 = 10^{-5}, \sigma_2 = 9\times 10^{3}, \sigma_3 = 5\times 10^{6}, \\ &\nu_1 = 0.5, \nu_2 = 0.05, \beta_3 = 600, \\ &\kappa_y = 1, \kappa_z = 10^{4}, \alpha = 6\times 10^{-5}. \end{aligned} \tag{P.11}$$

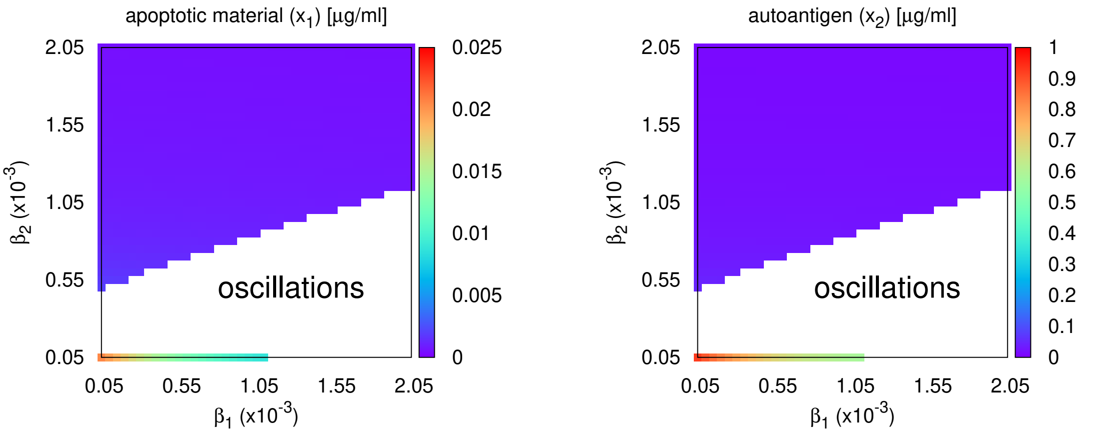

Figure S.16: Steady state values of the two types of antigen as function of β_1, β_2 in the stable steady state of type E_*. Remaining parameter values given in (P.11).

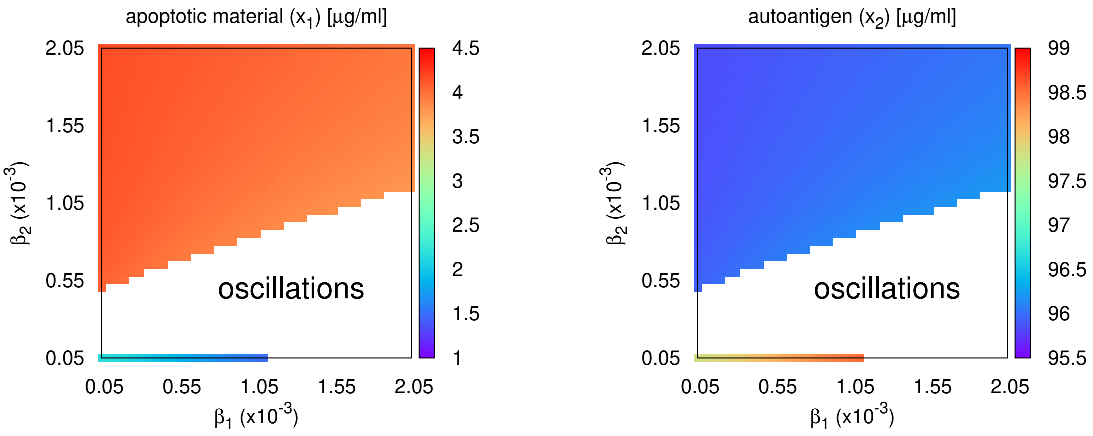

Figure S.17: Fractions of the two types of antigen as function of β_1, β_2 in the stable steady state of type E_*. Remaining parameter values given in (P.11).

Sensitivity of the fractions of the antigen to variation of β_1, β_2 and β_3 is done with the following parameter set:

$$\begin{aligned} &\mu_1 = \mu_2 = 12, \mu_3 = 0.95, \mu_4 = 0.2, \mu_5 = 8\times10^{-7}, \\ &\sigma_1 = 10^{-5}, \sigma_2 = 1.9\times10^4, \sigma_3 = 5\times10^6, \\ &\nu_1 = 0.05, \nu_2 = 0.5, \kappa_y = 1, \kappa_z = 10^4, \alpha = 9\times10^{-6}. \end{aligned} \tag{P.12}$$

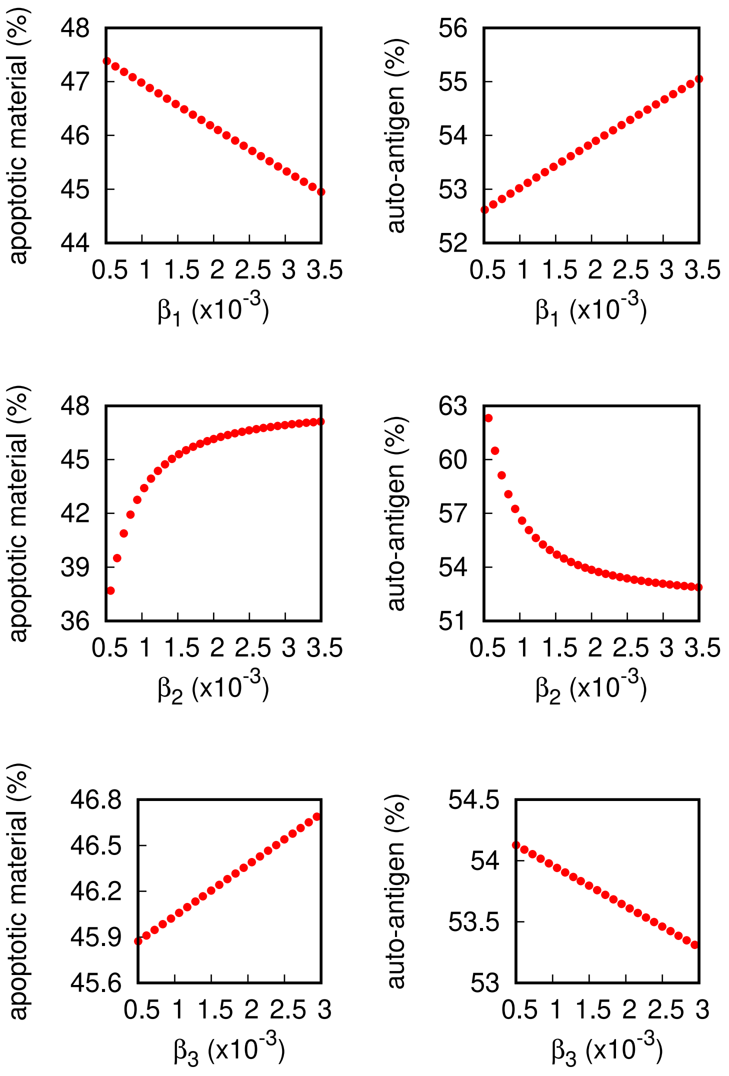

Figure S.18: Fractions of the two types of antigen as function of β_1 ($\beta_2 = 2\times10^{-3}, \beta_3 = 13.3\times10^4$ fixed), β_2 ($\beta_1 = 2\times10^{-3}, \beta_3 = 13.3\times10^4$ fixed), and β_3 ($\beta_1 = \beta_2 = 2\times10^{-3}$ fixed). The red solid dot represents the fractions at the stable coexistence equilibrium (parameters in (P.12)).

References

[1] A. F. Marée, R. Kublik, D. T. Finegood, and L. Edelstein-Keshet. "Modelling the onset of Type 1 diabetes: can impaired macrophage phagocytosis make the difference between health and disease?" In: Phil. Trans. R. Soc. A 364 (2006), pp. 1267–1282. DOI: 10.1098/rsta.2006.1769.

[2] N. Moise and A. Friedman. "Rheumatoid arthritis - a mathematical model". In: J. Theor. Biol. 461 (2019), pp. 17–33. DOI: 10.1016/j.jtbi.2018.10.039.

[3] A. E. M. van Nieuwenhuijze, T. van Lopik, R. J. T. Smeenk, and L. A. Aarden. "Time between onset of apoptosis and release of nucleosomes from apoptotic cells: putative implications for systemic lupus erythematosus". In: Ann. Rheum. Dis. 62 (2003), pp. 10–14. DOI: 10.1136/ard.62.1.10.

[4] A. A. Patel, F. Ginhoux, and S. Yona. "Monocytes, macrophages, dendritic cells and neutrophils: an update on lifespan kinetics in health and disease". In: Immunology 163 (2021), pp. 250–261. DOI: 10.1111/imm.13320.

[5] K. V. Rogers, S. W. Martin, I. Bhattacharya, R. S. P. Singh, and S. Nayak. "A dynamic quantitative systems pharmacology model of inflammatory bowel disease: part 2 – application to current therapies in Crohn's disease". In: Clin. Transl. Sci. 14 (2021), pp. 249–259. DOI: 10.1111/cts.12850.

[6] Y. Tatsukawa, W. Hsu, M. Yamada, J. Cologne, G. Suzuki, H. Yamamoto, K. Yamane, M. Akahoshi, S. Fujiwara, and N. Kohno. "White blood cell count, especially neutrophil count, as a predictor of hypertension in a Japanese population". In: Hypertens. Res. 31 (2008), pp. 1391–1397. DOI: 10.1291/hypres.31.1391.
